# Supplementary material for: Uncoupled evolution of the Polycomb system and deep origin of non-canonical PRC1
Source: Commun Biol. 2023 Nov 10;6:1144. doi: 10.1038/s42003-023-05501-x (PMC10638273; doi:10.1038/s42003-023-05501-x)
Supplement: Supplementary file 3 — Description of Supplementary Materials [file 42003_2023_5501_MOESM3_ESM.docx]

**Description of Additional Supplementary Files**

**File name:** Supplementary Dataset 1

**Description:** Proteome database. Table containing all eukaryotic species information from which sequences are used to construct the phylogenetic trees in SI Fig. S1A-H.

**File name:** Supplementary Dataset 2

**Description:** Manually curated HMM of RING1, PCGF, EZH, RBBP, EED, and SUZ12.

**File name:** Supplementary Dataset 3

**Description:** Materials that were used for the structural alignment. Includes TM align files, HHpred outputs and pdb files of excised C-terminal ß-hairpins.

**File name:** Supplementary Dataset 4

**Description:** Materials and Data that were used for the homology analyses of CBX, PHC, RYBP, and SUZ12. For CBX and RYBP, HMM profiles and HMMsearch outputs are provided. For PHC, SAM and MBT HMM profiles and searches, and newick files of our phylogenetic trees are provided. For SUZ12, HMM profile and multiple sequence alignment are provided.

**File name:** Supplementary Dataset 5

**Description:** Alphafold structures of putative sequences of RING and PCGF and the newly identified RYBP orthologs.

**File name:** Supplementary Dataset 6

**Description:** Blastoutputs of RING, PCGF, EZH, EED, and RBBP.

**File name:** Supplementary Dataset 7

**Description:** Multiple sequence alignments used to build phylogenetic trees and newick files for EZH, EED, RBBP, RING and PCGF.

**File name:** Supplementary Dataset 8

**Description:** Fasta sequence files of our orthologous groups of the core subunits of PRC1 and PRC2, and newly identified ncPRC1 accessory subunits.

**File name:** Supplementary Dataset 9

**Description:** Numerical source data for the pie charts presented in Figure 2.
