# Supplementary figures and images for: Uncoupled evolution of the Polycomb system and deep origin of non-canonical PRC1
Source: Commun Biol. 2023 Nov 10;6:1144. doi: 10.1038/s42003-023-05501-x (PMC10638273; doi:10.1038/s42003-023-05501-x)

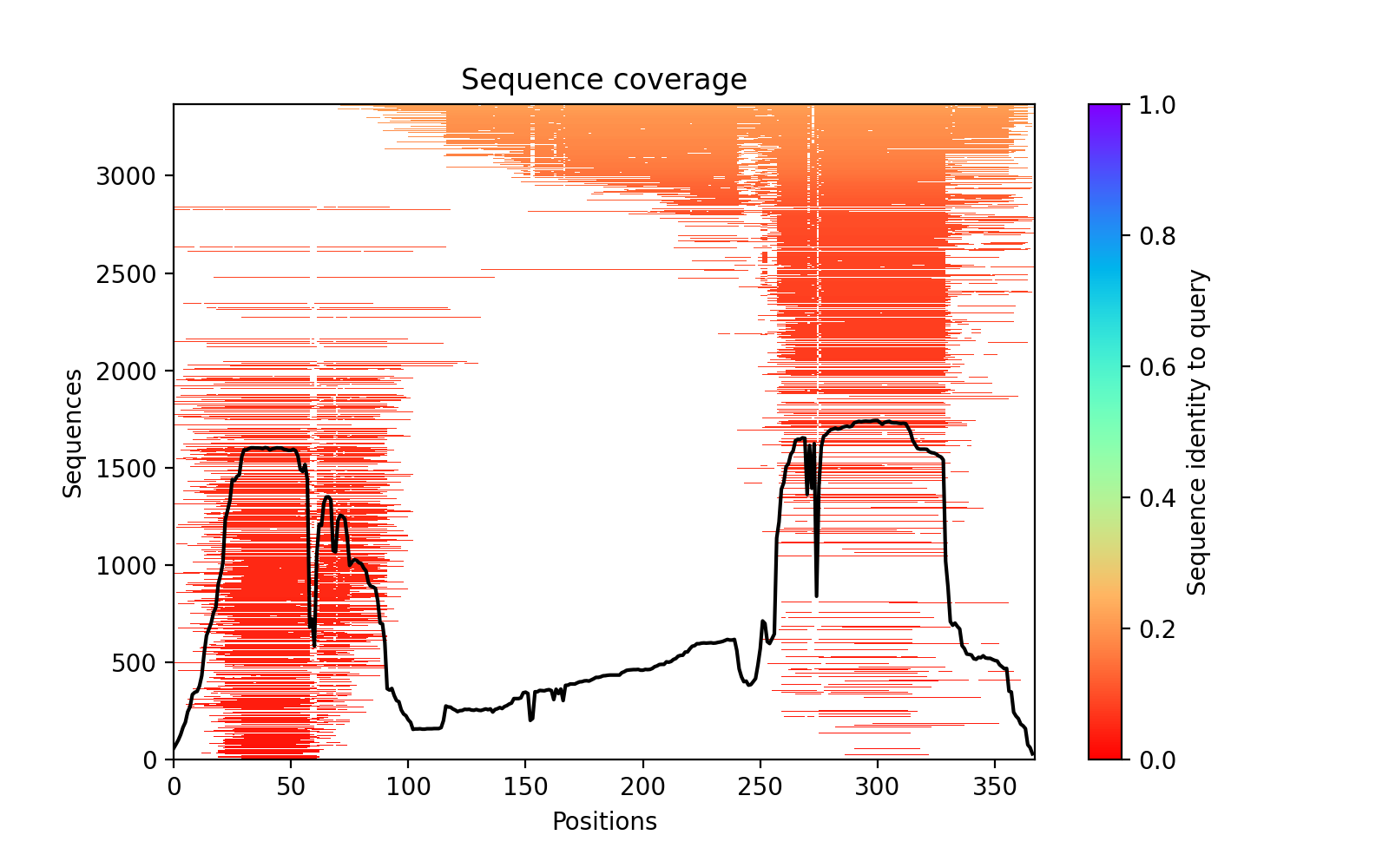

Supplement: Supplementary file 8 — Supplementary Data 5 [file 42003_2023_5501_MOESM8_ESM.zip › Supplementary Data 5/PARATL012597_SAM_527dc.result/PARATL012597_SAM_527dc_coverage.png]

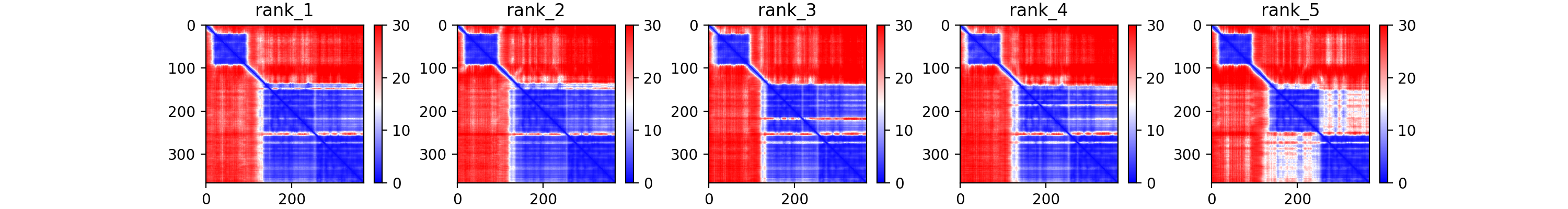

Supplement: Supplementary file 8 — Supplementary Data 5 [file 42003_2023_5501_MOESM8_ESM.zip › Supplementary Data 5/PARATL012597_SAM_527dc.result/PARATL012597_SAM_527dc_PAE.png]

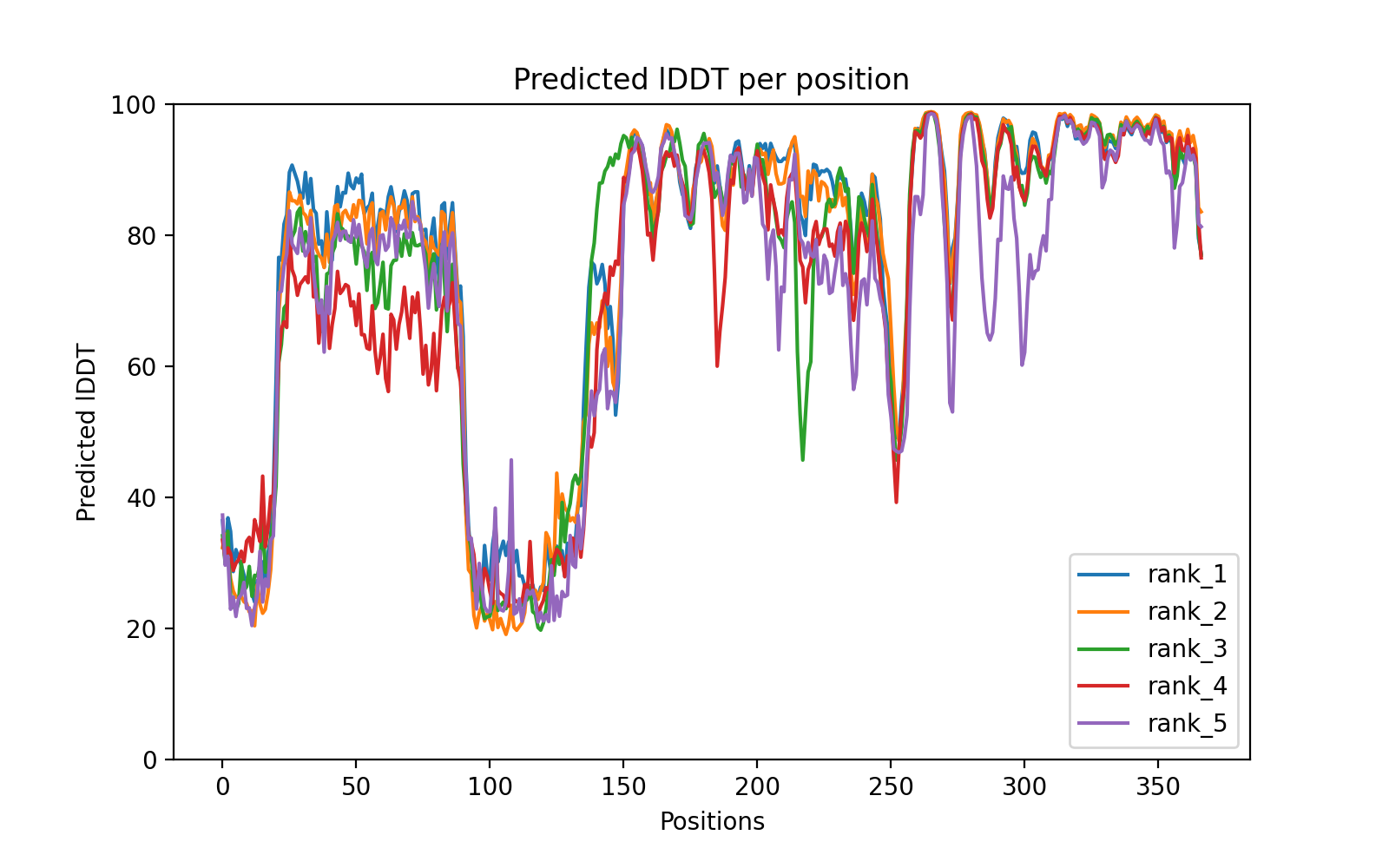

Supplement: Supplementary file 8 — Supplementary Data 5 [file 42003_2023_5501_MOESM8_ESM.zip › Supplementary Data 5/PARATL012597_SAM_527dc.result/PARATL012597_SAM_527dc_plddt.png]

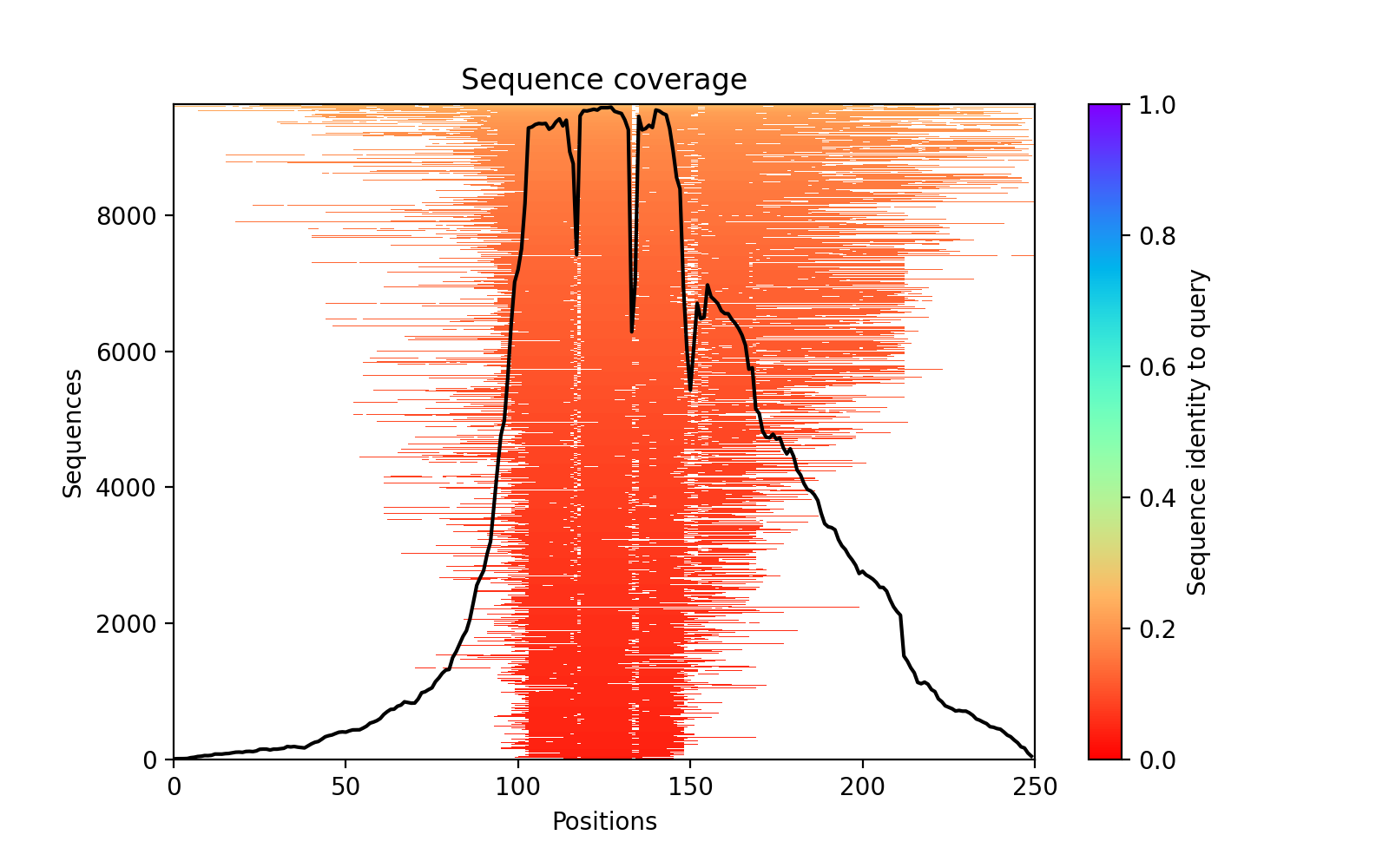

Supplement: Supplementary file 8 — Supplementary Data 5 [file 42003_2023_5501_MOESM8_ESM.zip › Supplementary Data 5/PCGF_putative_checks/PCGF_alphafold_remainedputative/ANCTWI007962_PCGF_8bd3b.result/ANCTWI007962_PCGF_8bd3b_coverage.png]

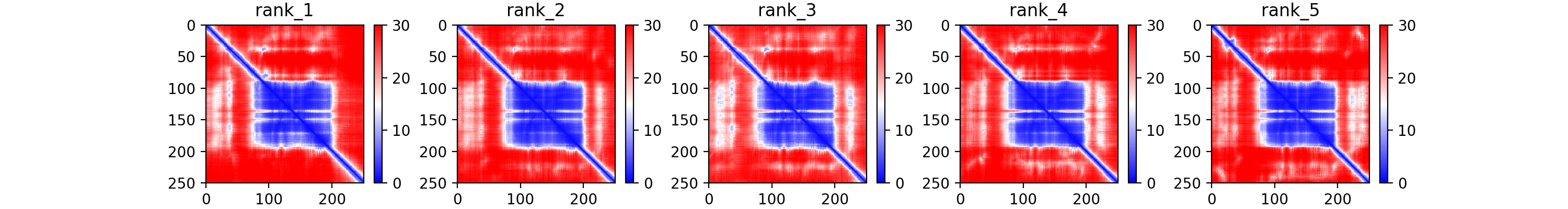

Supplement: Supplementary file 8 — Supplementary Data 5 [file 42003_2023_5501_MOESM8_ESM.zip › Supplementary Data 5/PCGF_putative_checks/PCGF_alphafold_remainedputative/ANCTWI007962_PCGF_8bd3b.result/ANCTWI007962_PCGF_8bd3b_PAE.png]

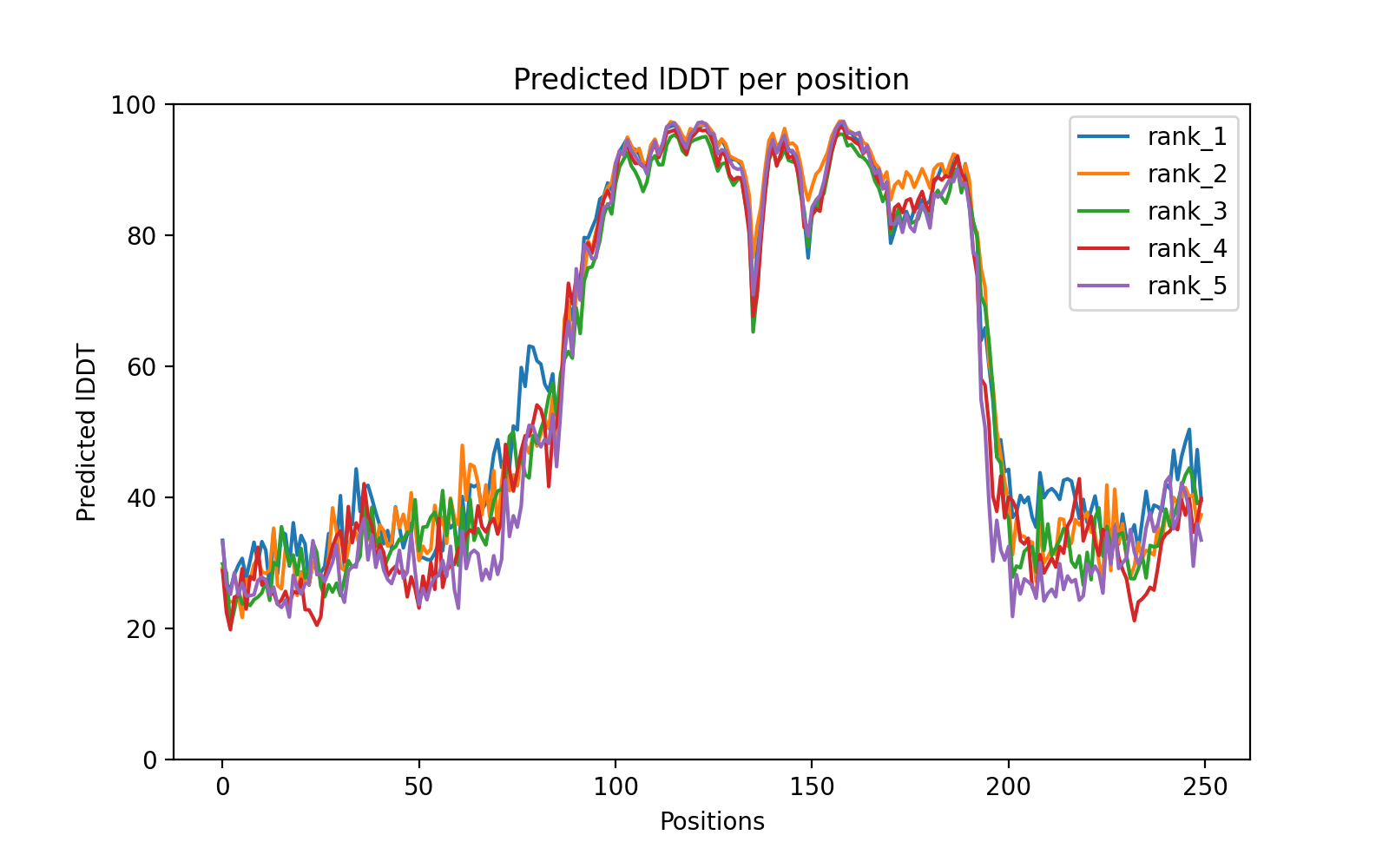

Supplement: Supplementary file 8 — Supplementary Data 5 [file 42003_2023_5501_MOESM8_ESM.zip › Supplementary Data 5/PCGF_putative_checks/PCGF_alphafold_remainedputative/ANCTWI007962_PCGF_8bd3b.result/ANCTWI007962_PCGF_8bd3b_plddt.png]

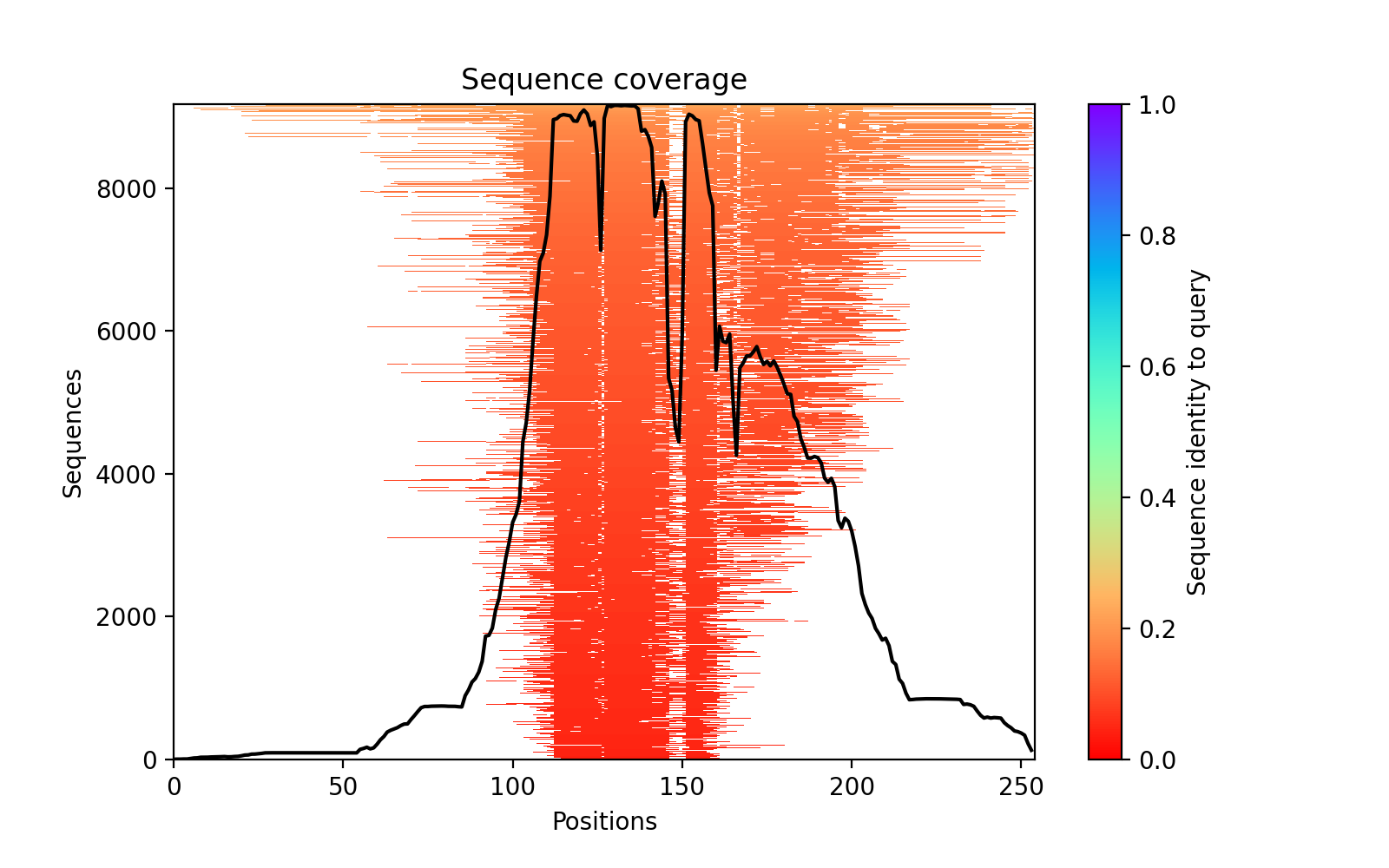

Supplement: Supplementary file 8 — Supplementary Data 5 [file 42003_2023_5501_MOESM8_ESM.zip › Supplementary Data 5/PCGF_putative_checks/PCGF_alphafold_remainedputative/BREMOT008734_PCGF_a3129.result/BREMOT008734_PCGF_a3129_coverage.png]

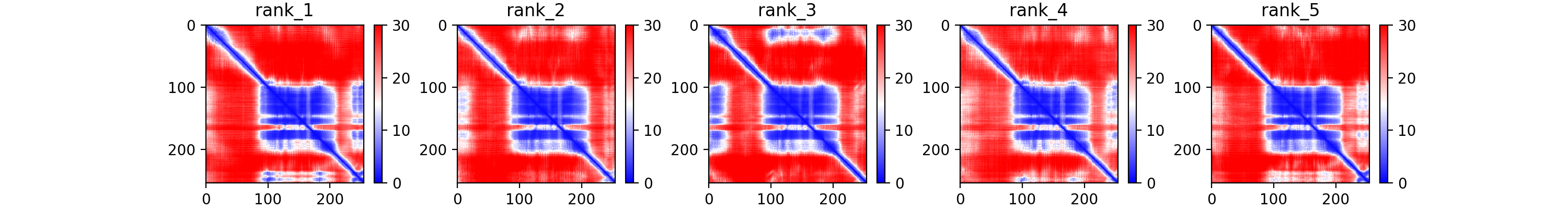

Supplement: Supplementary file 8 — Supplementary Data 5 [file 42003_2023_5501_MOESM8_ESM.zip › Supplementary Data 5/PCGF_putative_checks/PCGF_alphafold_remainedputative/BREMOT008734_PCGF_a3129.result/BREMOT008734_PCGF_a3129_PAE.png]

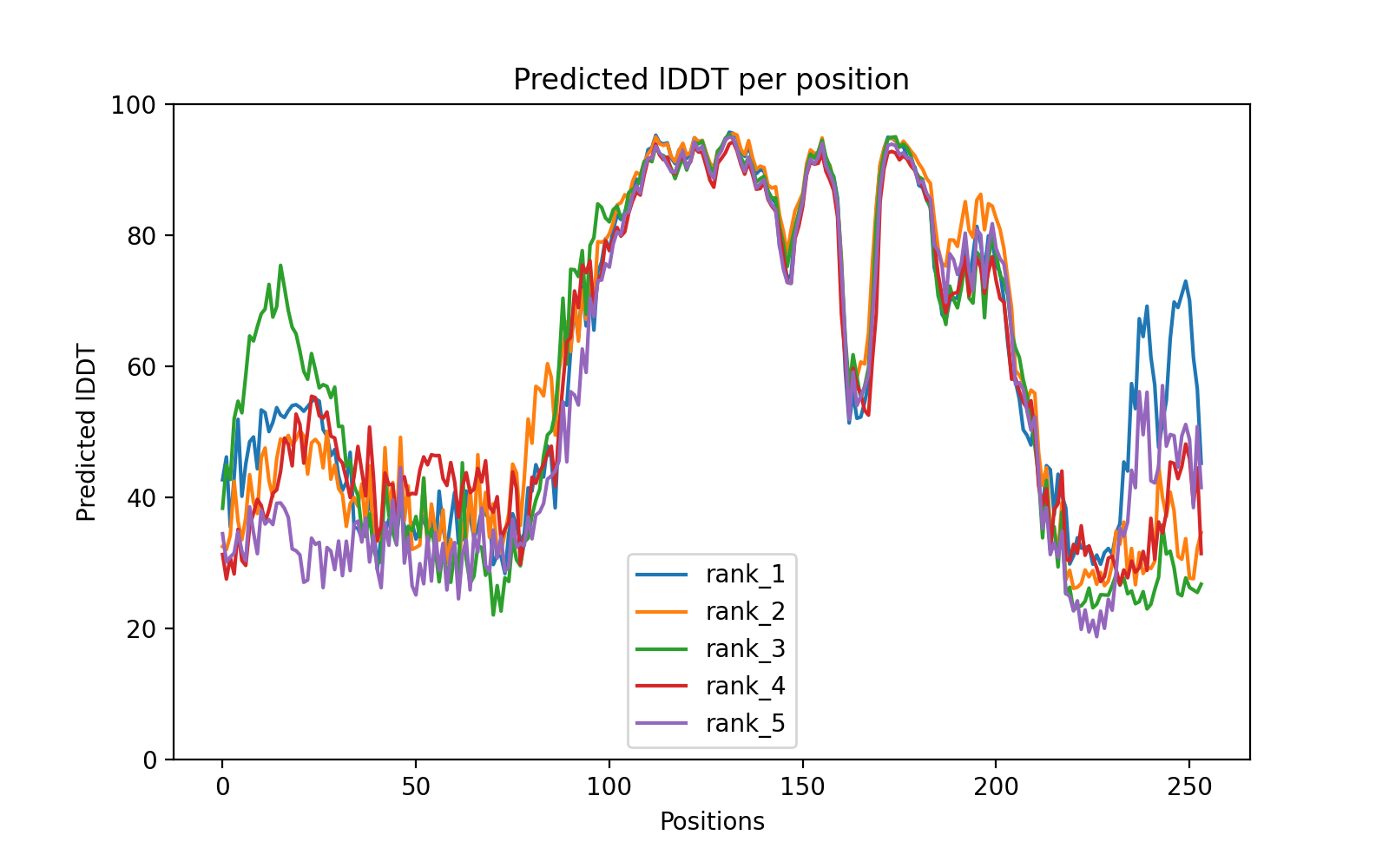

Supplement: Supplementary file 8 — Supplementary Data 5 [file 42003_2023_5501_MOESM8_ESM.zip › Supplementary Data 5/PCGF_putative_checks/PCGF_alphafold_remainedputative/BREMOT008734_PCGF_a3129.result/BREMOT008734_PCGF_a3129_plddt.png]

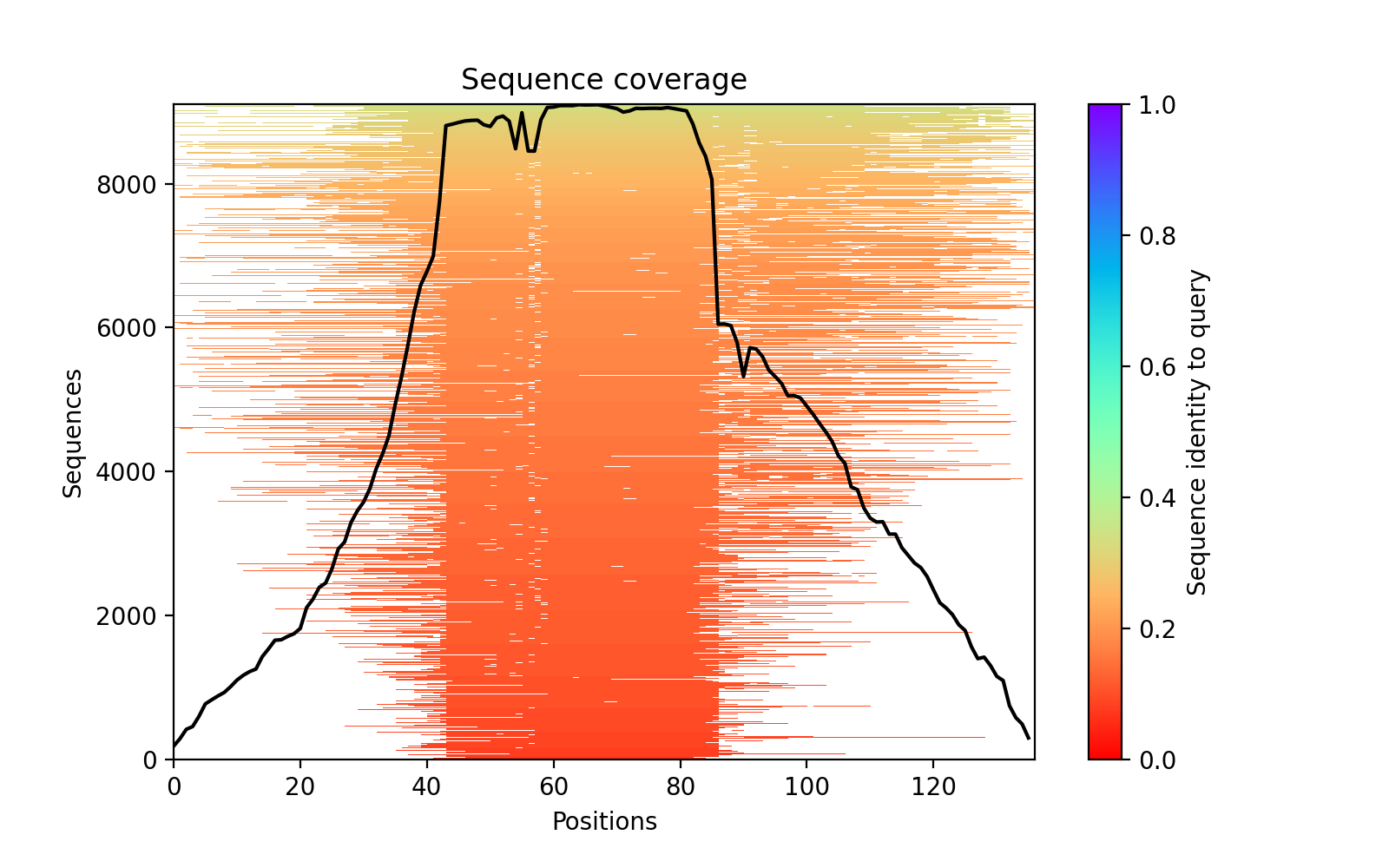

Supplement: Supplementary file 8 — Supplementary Data 5 [file 42003_2023_5501_MOESM8_ESM.zip › Supplementary Data 5/PCGF_putative_checks/PCGF_alphafold_remainedputative/CHOSPE030776_PCGF_fcfb5.result/CHOSPE030776_PCGF_fcfb5_coverage.png]

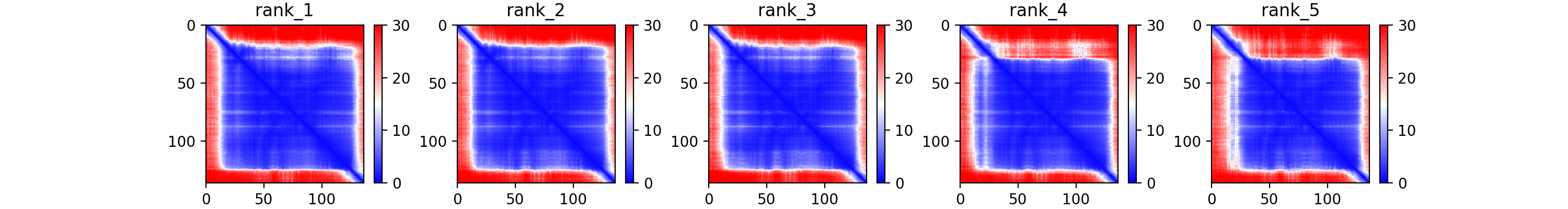

Supplement: Supplementary file 8 — Supplementary Data 5 [file 42003_2023_5501_MOESM8_ESM.zip › Supplementary Data 5/PCGF_putative_checks/PCGF_alphafold_remainedputative/CHOSPE030776_PCGF_fcfb5.result/CHOSPE030776_PCGF_fcfb5_PAE.png]

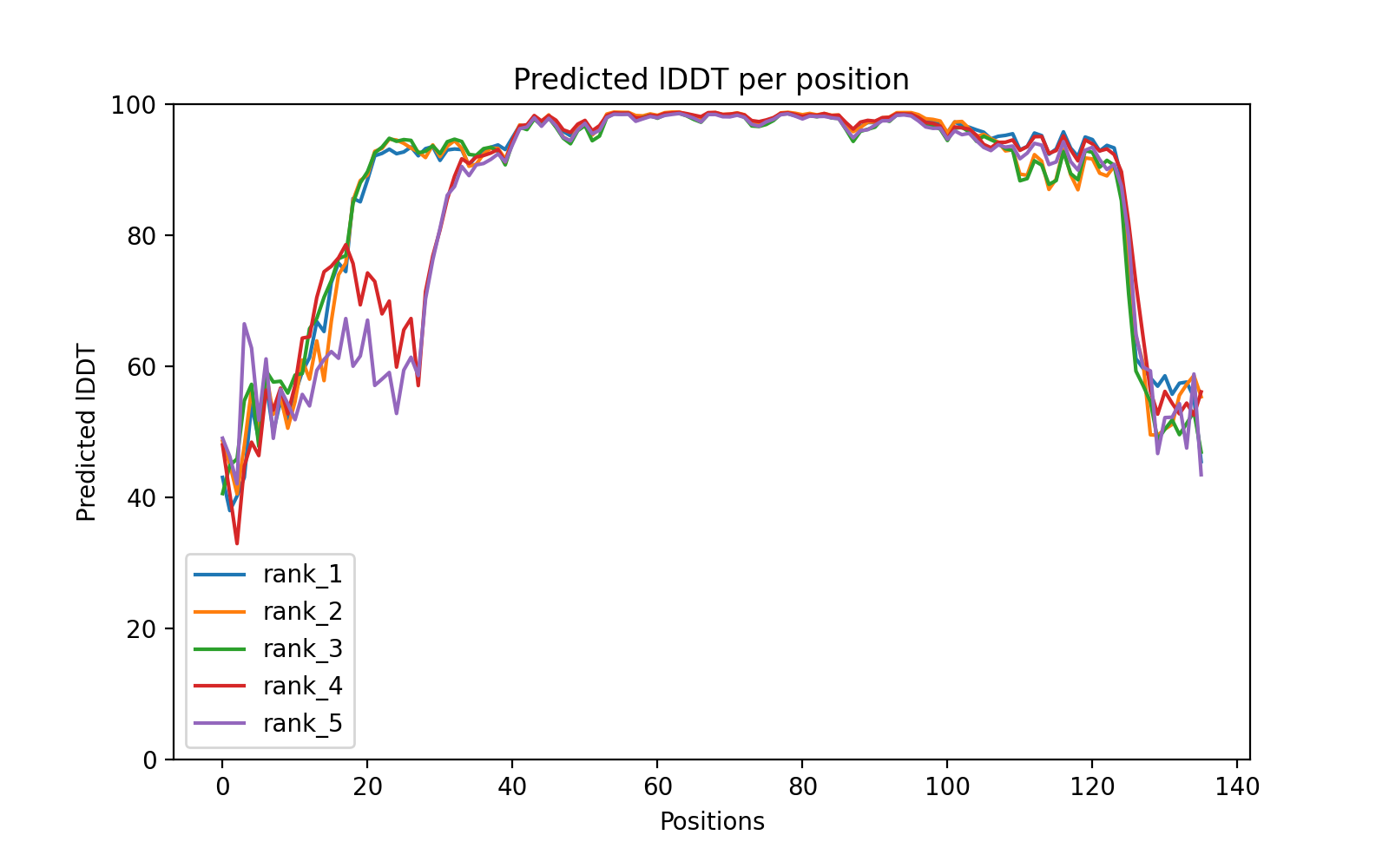

Supplement: Supplementary file 8 — Supplementary Data 5 [file 42003_2023_5501_MOESM8_ESM.zip › Supplementary Data 5/PCGF_putative_checks/PCGF_alphafold_remainedputative/CHOSPE030776_PCGF_fcfb5.result/CHOSPE030776_PCGF_fcfb5_plddt.png]

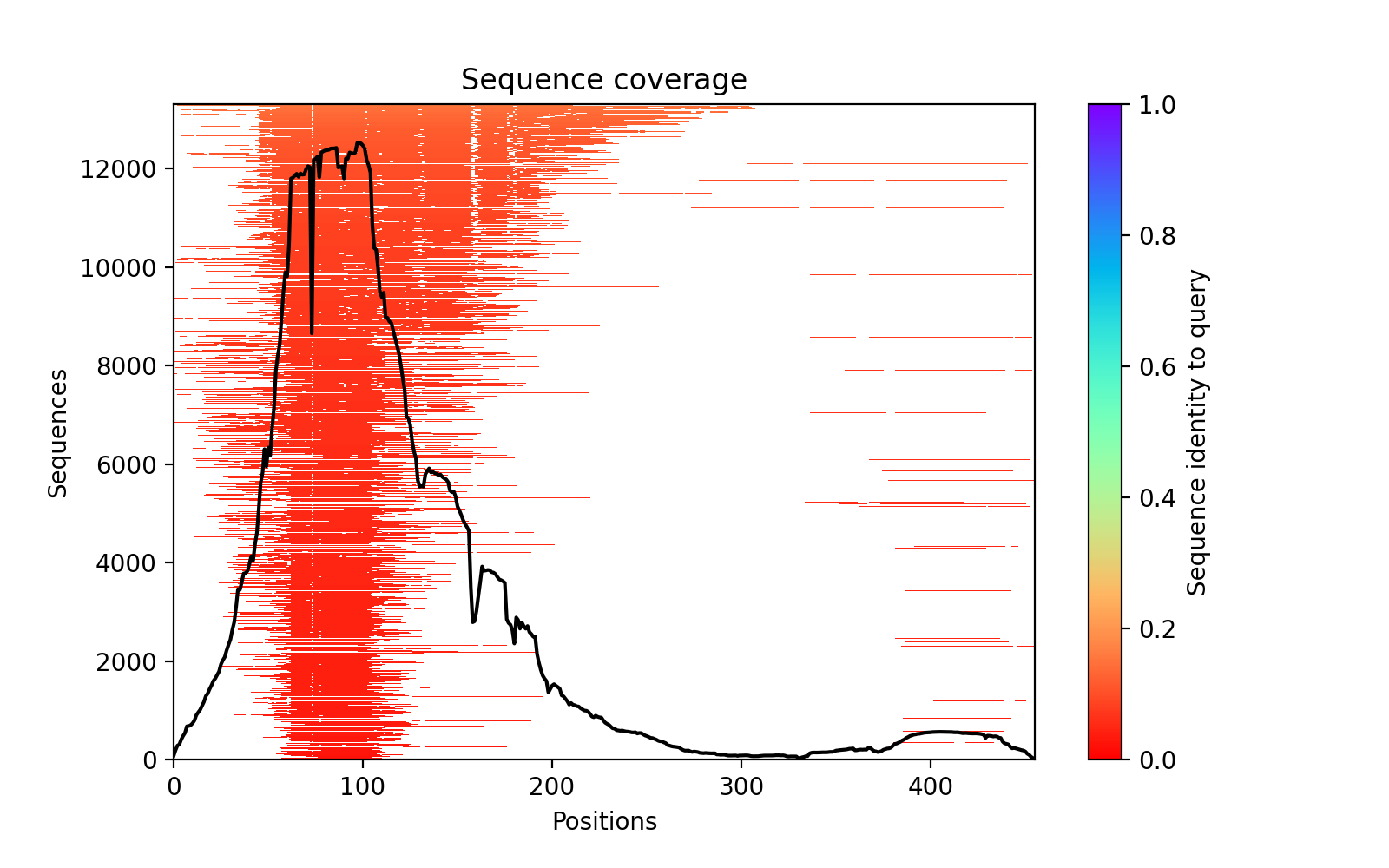

Supplement: Supplementary file 8 — Supplementary Data 5 [file 42003_2023_5501_MOESM8_ESM.zip › Supplementary Data 5/PCGF_putative_checks/PCGF_alphafold_remainedputative/HEMPHA007334_PCGF_68d35.result/HEMPHA007334_PCGF_68d35_coverage.png]

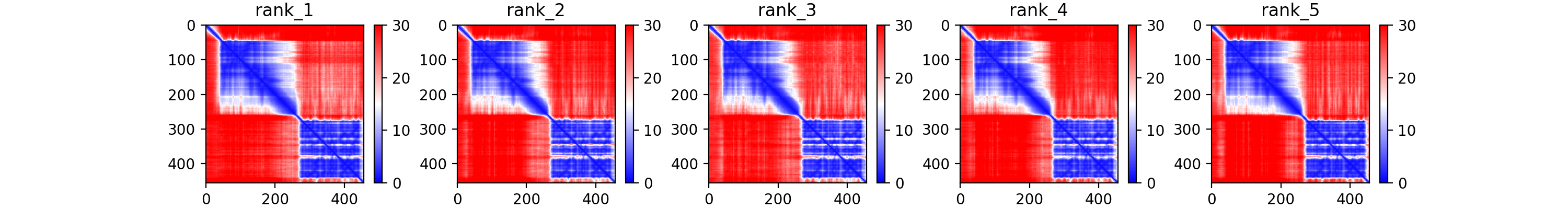

Supplement: Supplementary file 8 — Supplementary Data 5 [file 42003_2023_5501_MOESM8_ESM.zip › Supplementary Data 5/PCGF_putative_checks/PCGF_alphafold_remainedputative/HEMPHA007334_PCGF_68d35.result/HEMPHA007334_PCGF_68d35_PAE.png]

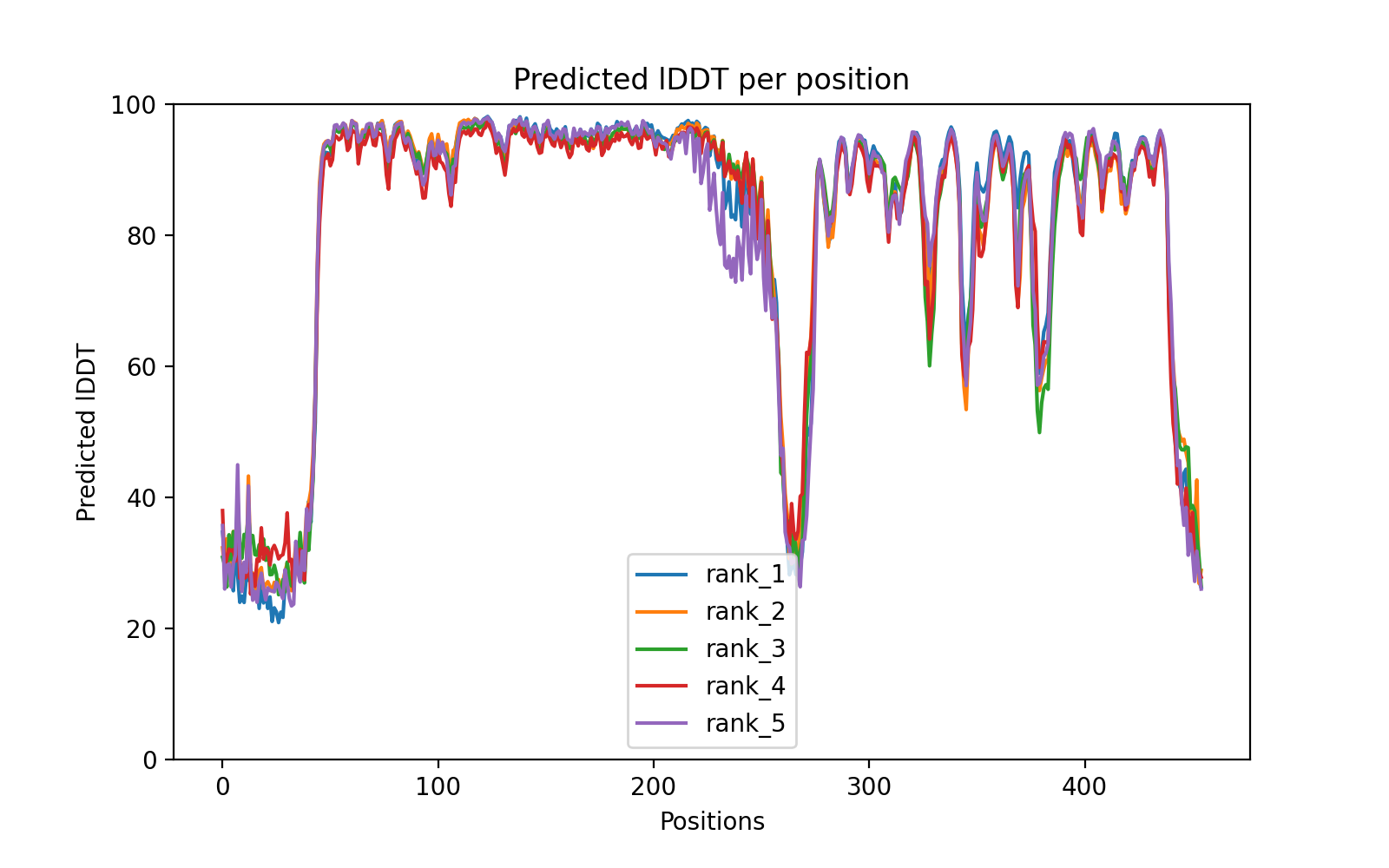

Supplement: Supplementary file 8 — Supplementary Data 5 [file 42003_2023_5501_MOESM8_ESM.zip › Supplementary Data 5/PCGF_putative_checks/PCGF_alphafold_remainedputative/HEMPHA007334_PCGF_68d35.result/HEMPHA007334_PCGF_68d35_plddt.png]

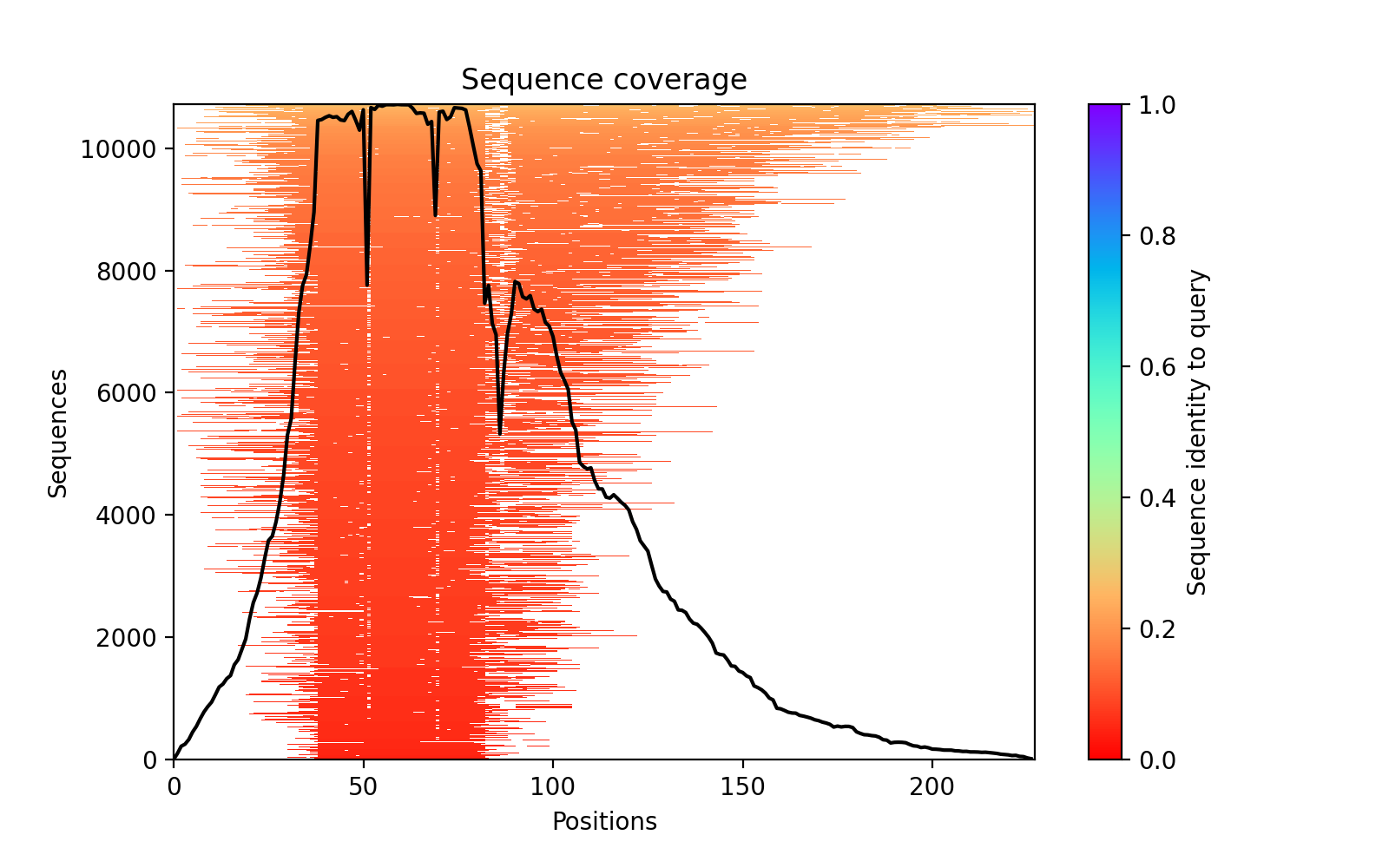

Supplement: Supplementary file 8 — Supplementary Data 5 [file 42003_2023_5501_MOESM8_ESM.zip › Supplementary Data 5/PCGF_putative_checks/PCGF_alphafold_remainedputative/NEPPYR005209_PCGF_19d22.result/NEPPYR005209_PCGF_19d22_coverage.png]

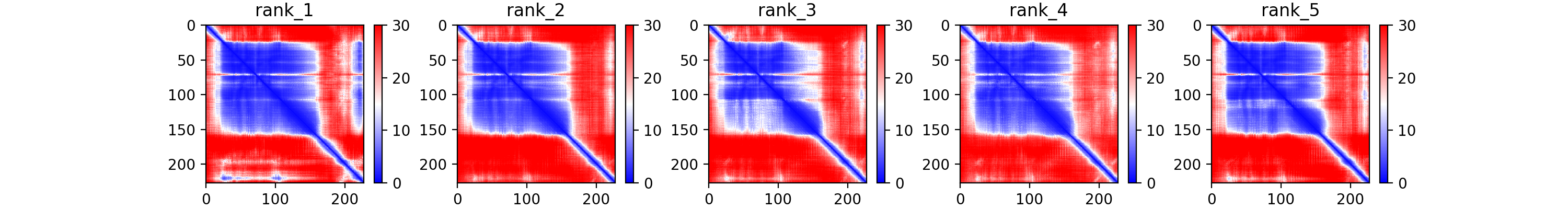

Supplement: Supplementary file 8 — Supplementary Data 5 [file 42003_2023_5501_MOESM8_ESM.zip › Supplementary Data 5/PCGF_putative_checks/PCGF_alphafold_remainedputative/NEPPYR005209_PCGF_19d22.result/NEPPYR005209_PCGF_19d22_PAE.png]

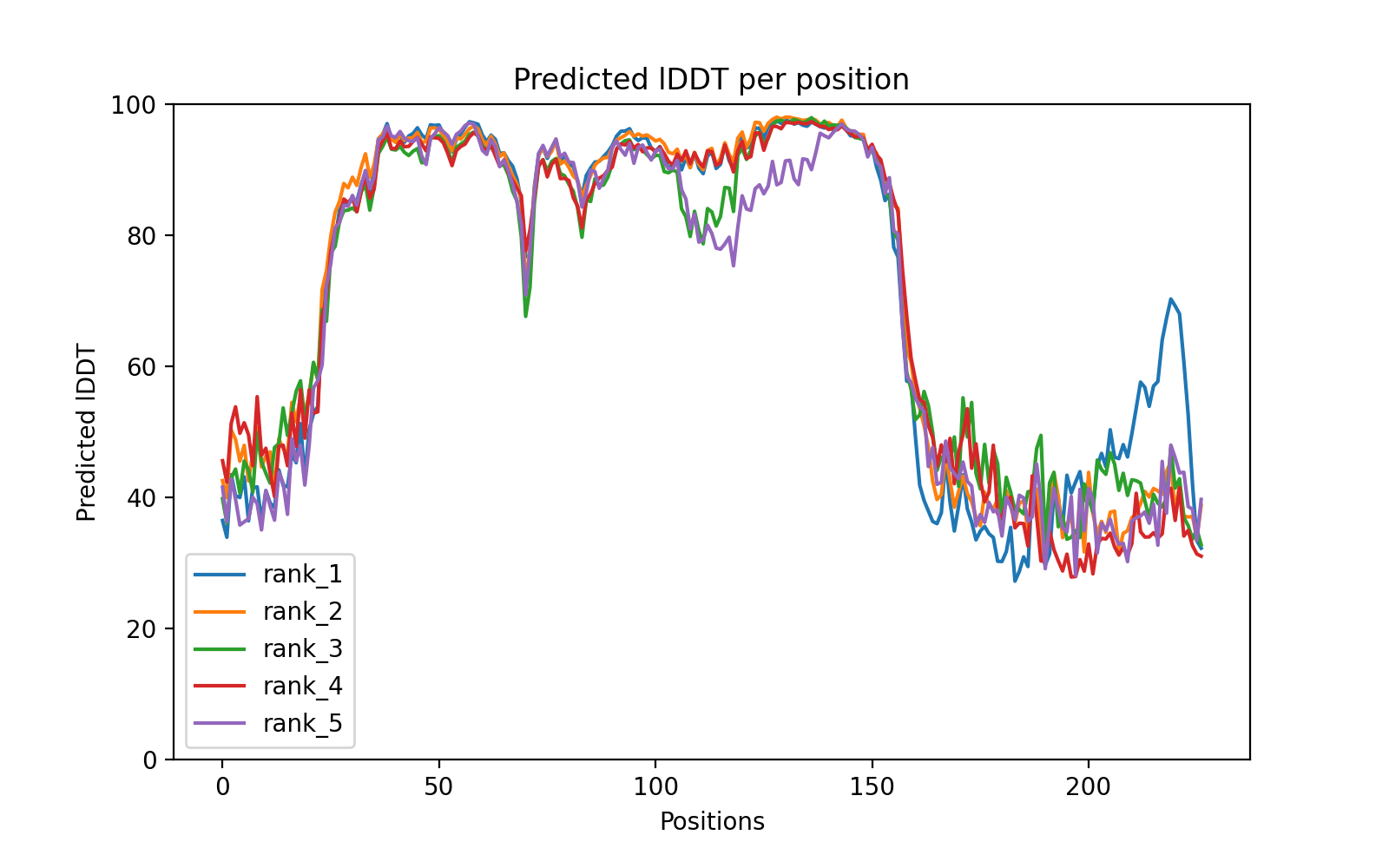

Supplement: Supplementary file 8 — Supplementary Data 5 [file 42003_2023_5501_MOESM8_ESM.zip › Supplementary Data 5/PCGF_putative_checks/PCGF_alphafold_remainedputative/NEPPYR005209_PCGF_19d22.result/NEPPYR005209_PCGF_19d22_plddt.png]

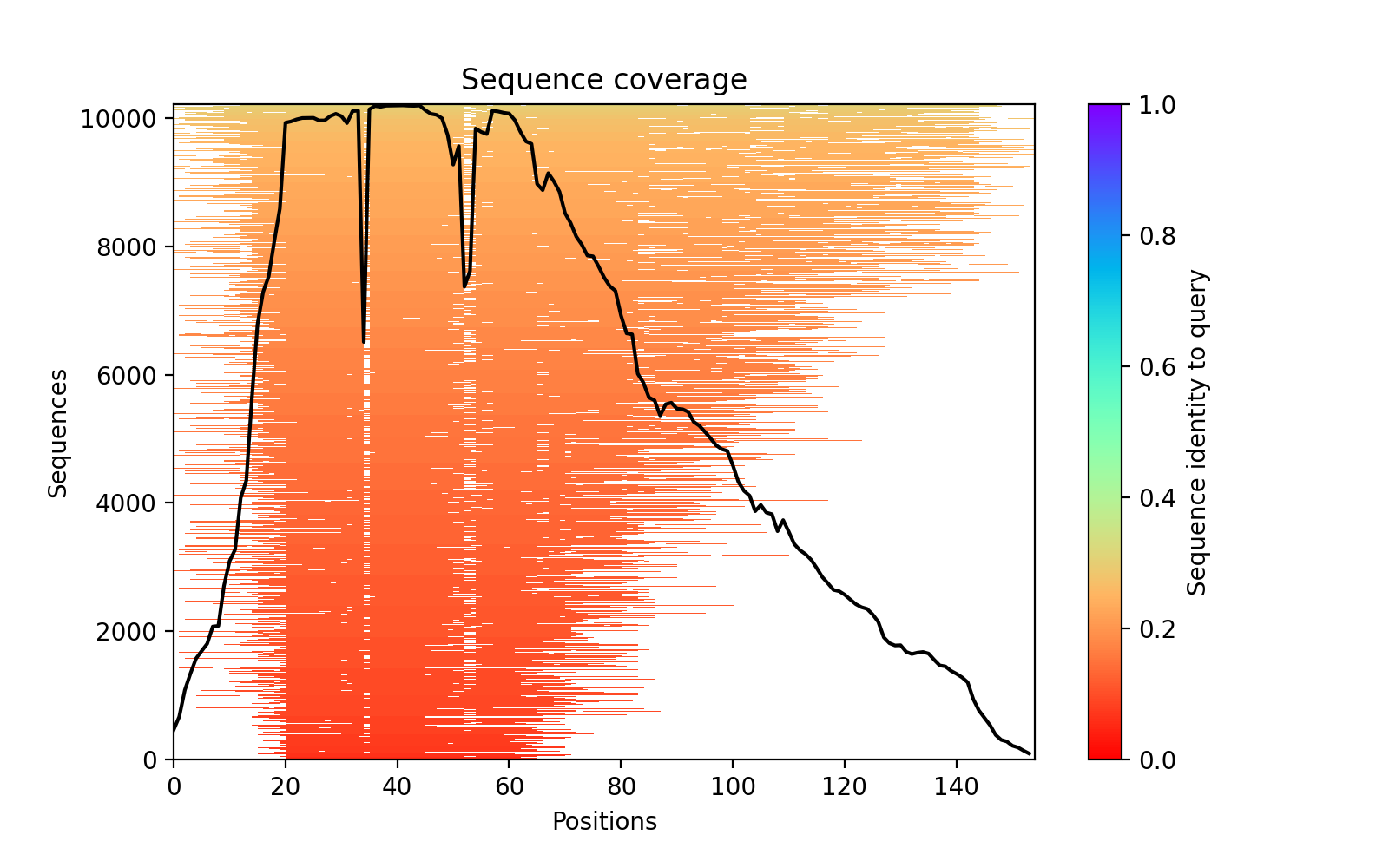

Supplement: Supplementary file 8 — Supplementary Data 5 [file 42003_2023_5501_MOESM8_ESM.zip › Supplementary Data 5/PCGF_putative_checks/PCGF_alphafold_remainedputative/NUTLON020672_PCGF_625f3.result/NUTLON020672_PCGF_625f3_coverage.png]

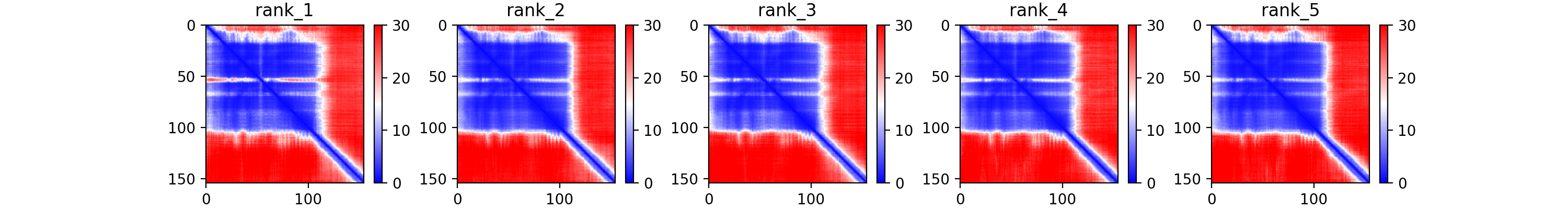

Supplement: Supplementary file 8 — Supplementary Data 5 [file 42003_2023_5501_MOESM8_ESM.zip › Supplementary Data 5/PCGF_putative_checks/PCGF_alphafold_remainedputative/NUTLON020672_PCGF_625f3.result/NUTLON020672_PCGF_625f3_PAE.png]

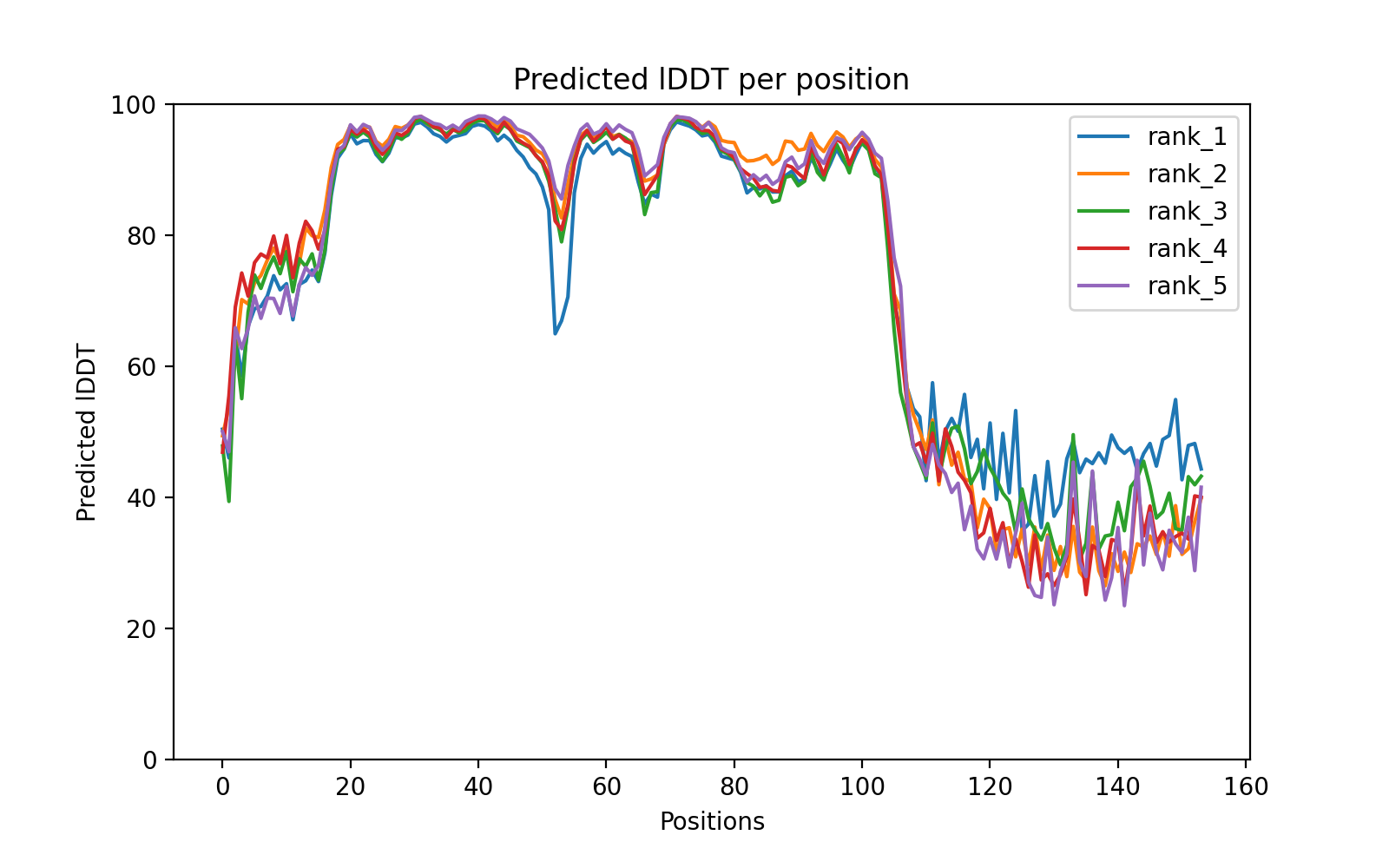

Supplement: Supplementary file 8 — Supplementary Data 5 [file 42003_2023_5501_MOESM8_ESM.zip › Supplementary Data 5/PCGF_putative_checks/PCGF_alphafold_remainedputative/NUTLON020672_PCGF_625f3.result/NUTLON020672_PCGF_625f3_plddt.png]

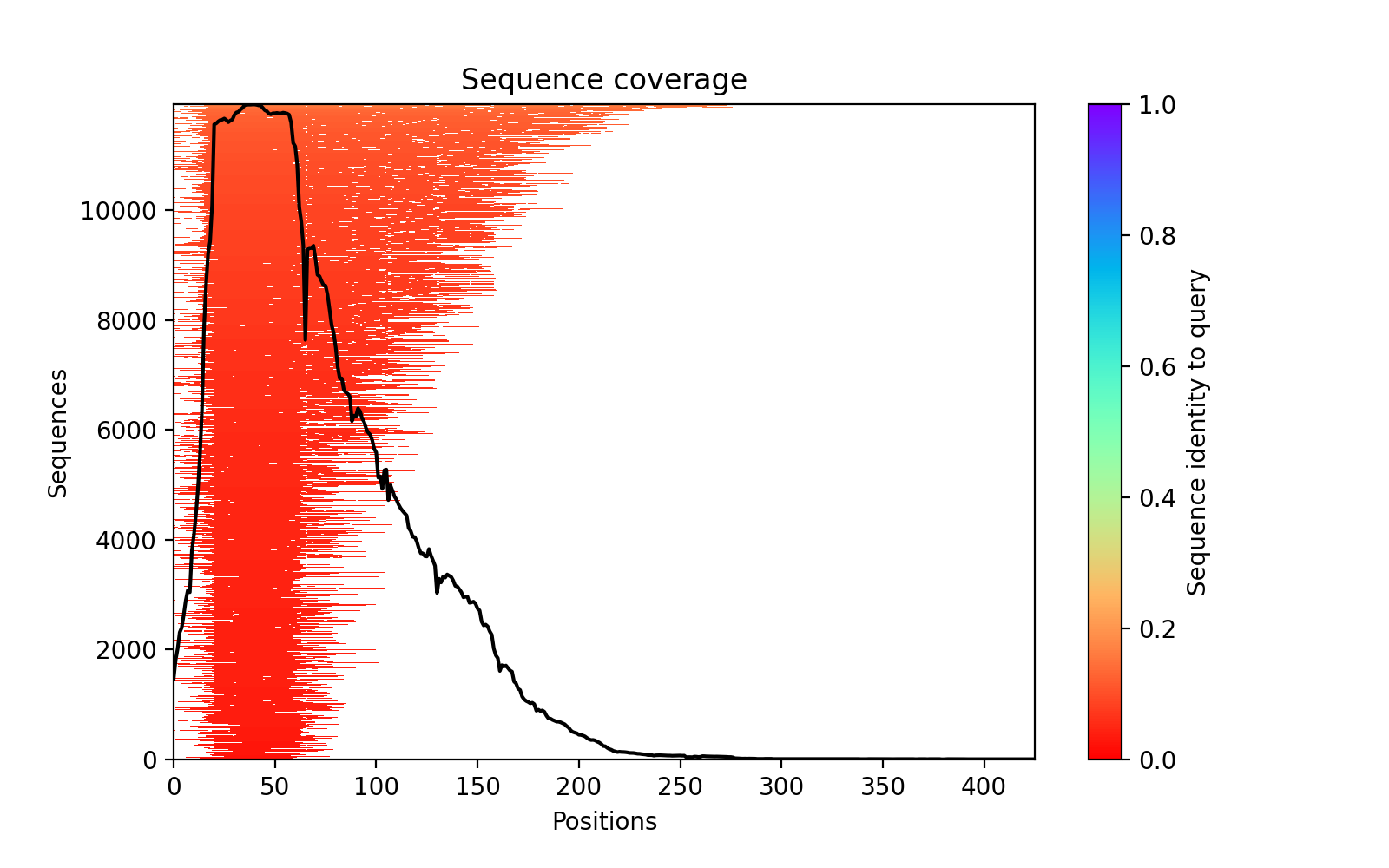

Supplement: Supplementary file 8 — Supplementary Data 5 [file 42003_2023_5501_MOESM8_ESM.zip › Supplementary Data 5/PCGF_putative_checks/PCGF_alphafold_remainedputative/PARTET031207_PCGF_63f22.result/PARTET031207_PCGF_63f22_coverage.png]

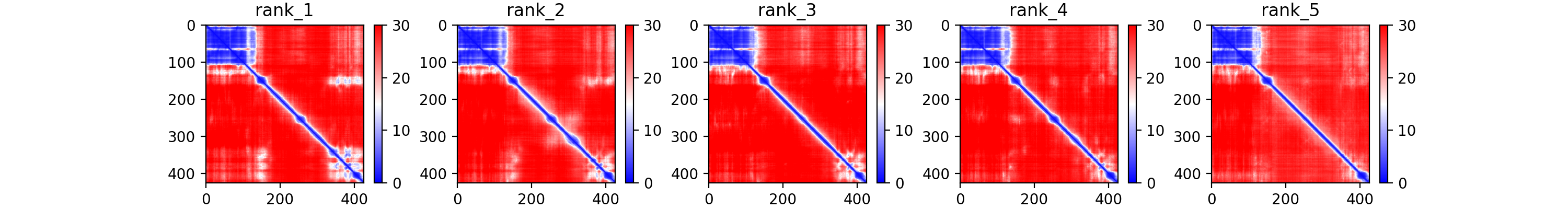

Supplement: Supplementary file 8 — Supplementary Data 5 [file 42003_2023_5501_MOESM8_ESM.zip › Supplementary Data 5/PCGF_putative_checks/PCGF_alphafold_remainedputative/PARTET031207_PCGF_63f22.result/PARTET031207_PCGF_63f22_PAE.png]

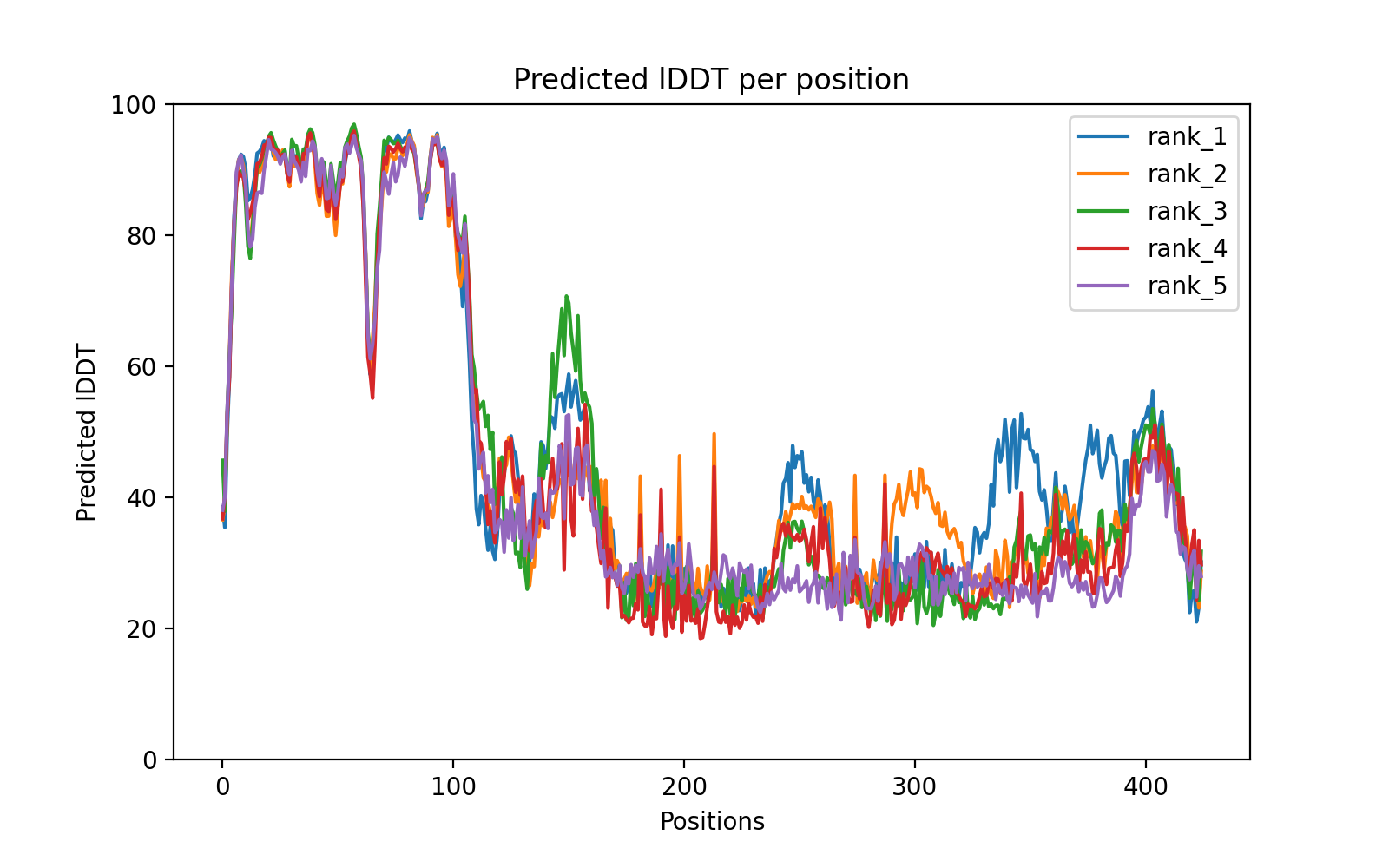

Supplement: Supplementary file 8 — Supplementary Data 5 [file 42003_2023_5501_MOESM8_ESM.zip › Supplementary Data 5/PCGF_putative_checks/PCGF_alphafold_remainedputative/PARTET031207_PCGF_63f22.result/PARTET031207_PCGF_63f22_plddt.png]

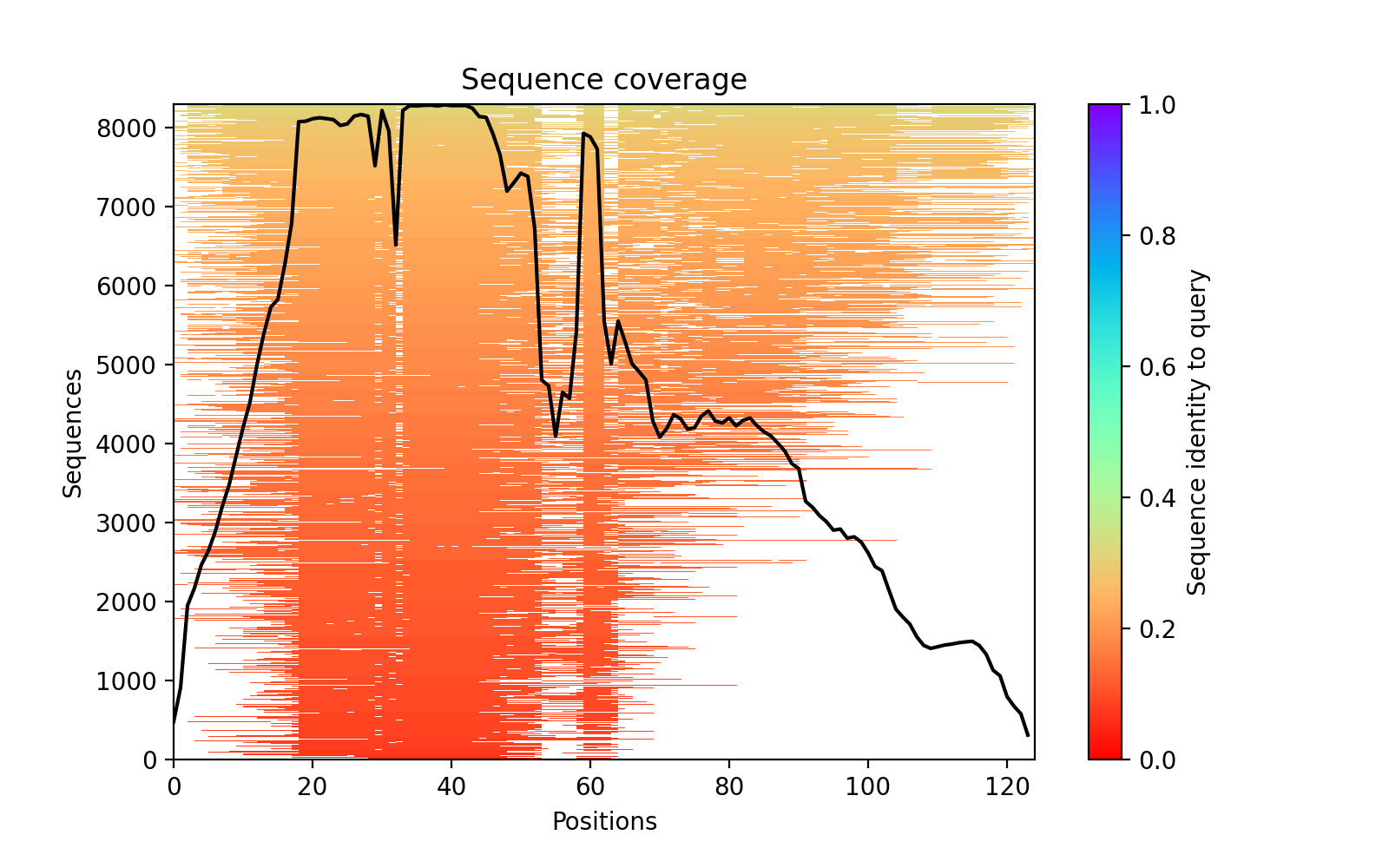

Supplement: Supplementary file 8 — Supplementary Data 5 [file 42003_2023_5501_MOESM8_ESM.zip › Supplementary Data 5/PCGF_putative_checks/PCGF_alphafold_remainedputative/PAUCHR028915_PCGF_c2bfc.result/PAUCHR028915_PCGF_c2bfc_coverage.png]

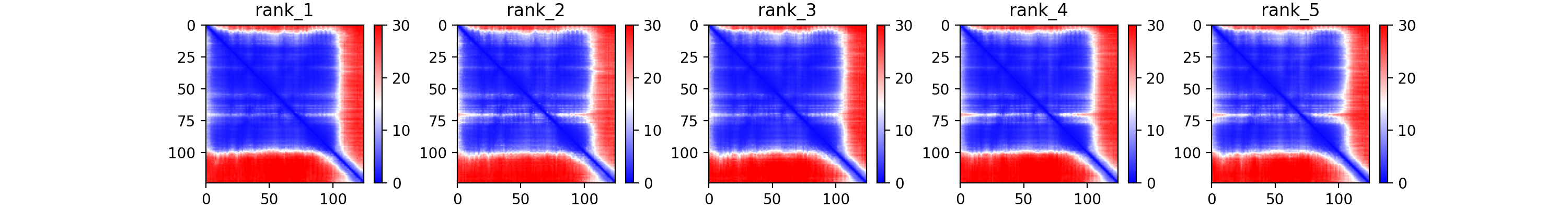

Supplement: Supplementary file 8 — Supplementary Data 5 [file 42003_2023_5501_MOESM8_ESM.zip › Supplementary Data 5/PCGF_putative_checks/PCGF_alphafold_remainedputative/PAUCHR028915_PCGF_c2bfc.result/PAUCHR028915_PCGF_c2bfc_PAE.png]

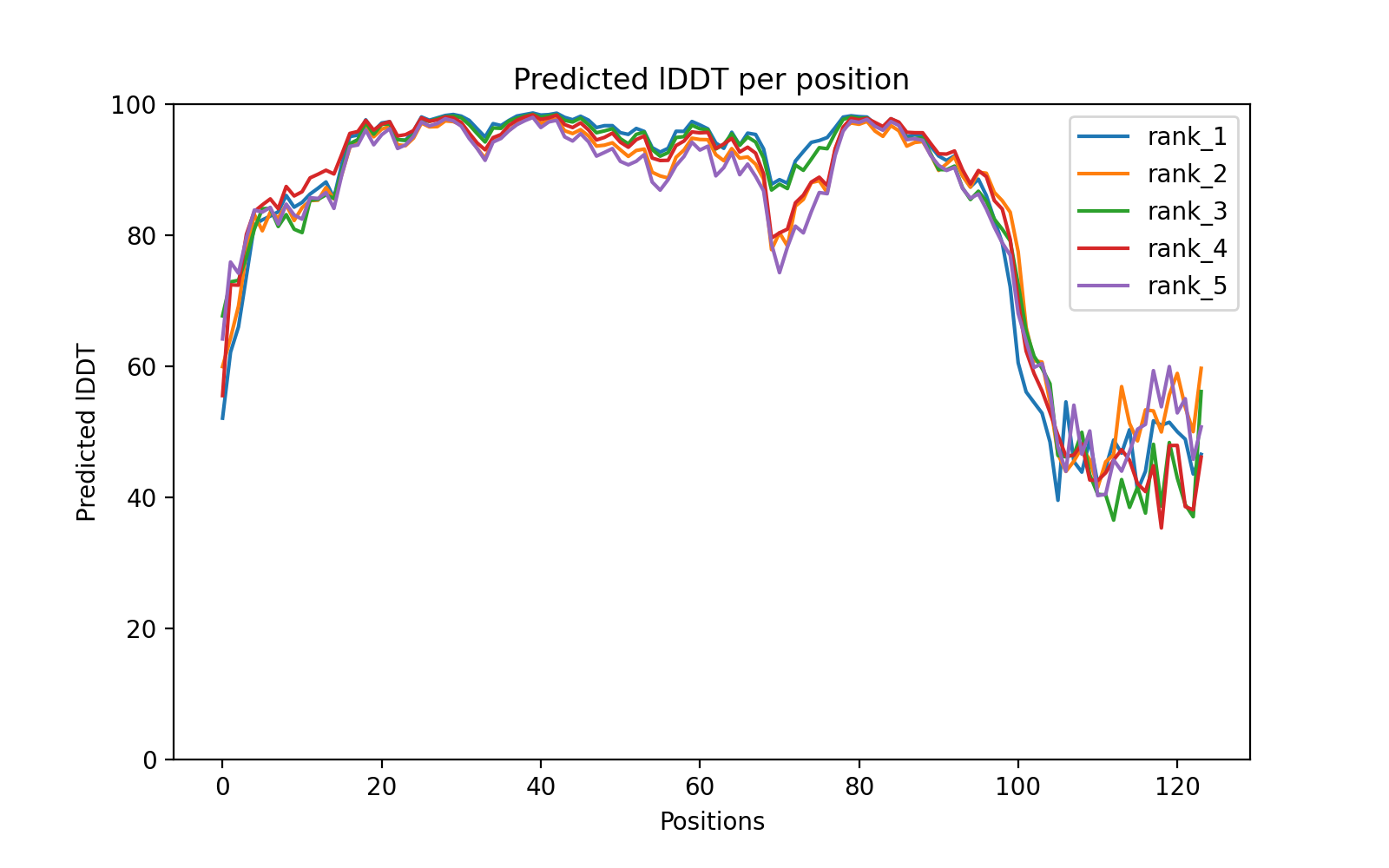

Supplement: Supplementary file 8 — Supplementary Data 5 [file 42003_2023_5501_MOESM8_ESM.zip › Supplementary Data 5/PCGF_putative_checks/PCGF_alphafold_remainedputative/PAUCHR028915_PCGF_c2bfc.result/PAUCHR028915_PCGF_c2bfc_plddt.png]

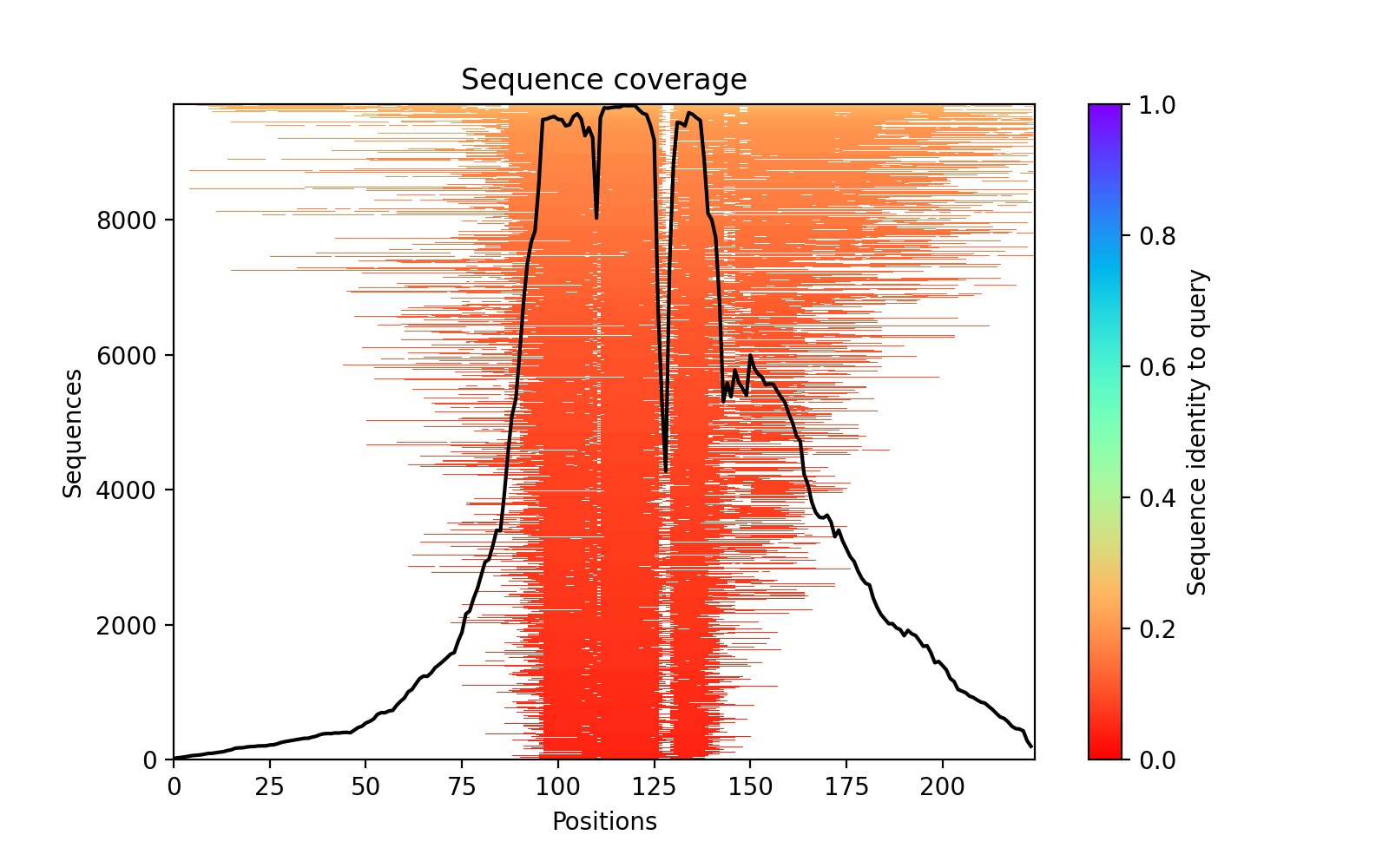

Supplement: Supplementary file 8 — Supplementary Data 5 [file 42003_2023_5501_MOESM8_ESM.zip › Supplementary Data 5/PCGF_putative_checks/PCGF_alphafold_remainedputative/PYGBIF005603_PCGF_458fe.result/PYGBIF005603_PCGF_458fe_coverage.png]

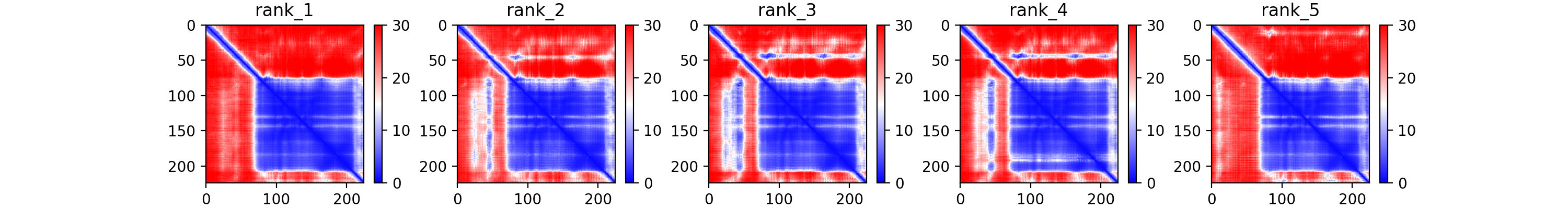

Supplement: Supplementary file 8 — Supplementary Data 5 [file 42003_2023_5501_MOESM8_ESM.zip › Supplementary Data 5/PCGF_putative_checks/PCGF_alphafold_remainedputative/PYGBIF005603_PCGF_458fe.result/PYGBIF005603_PCGF_458fe_PAE.png]

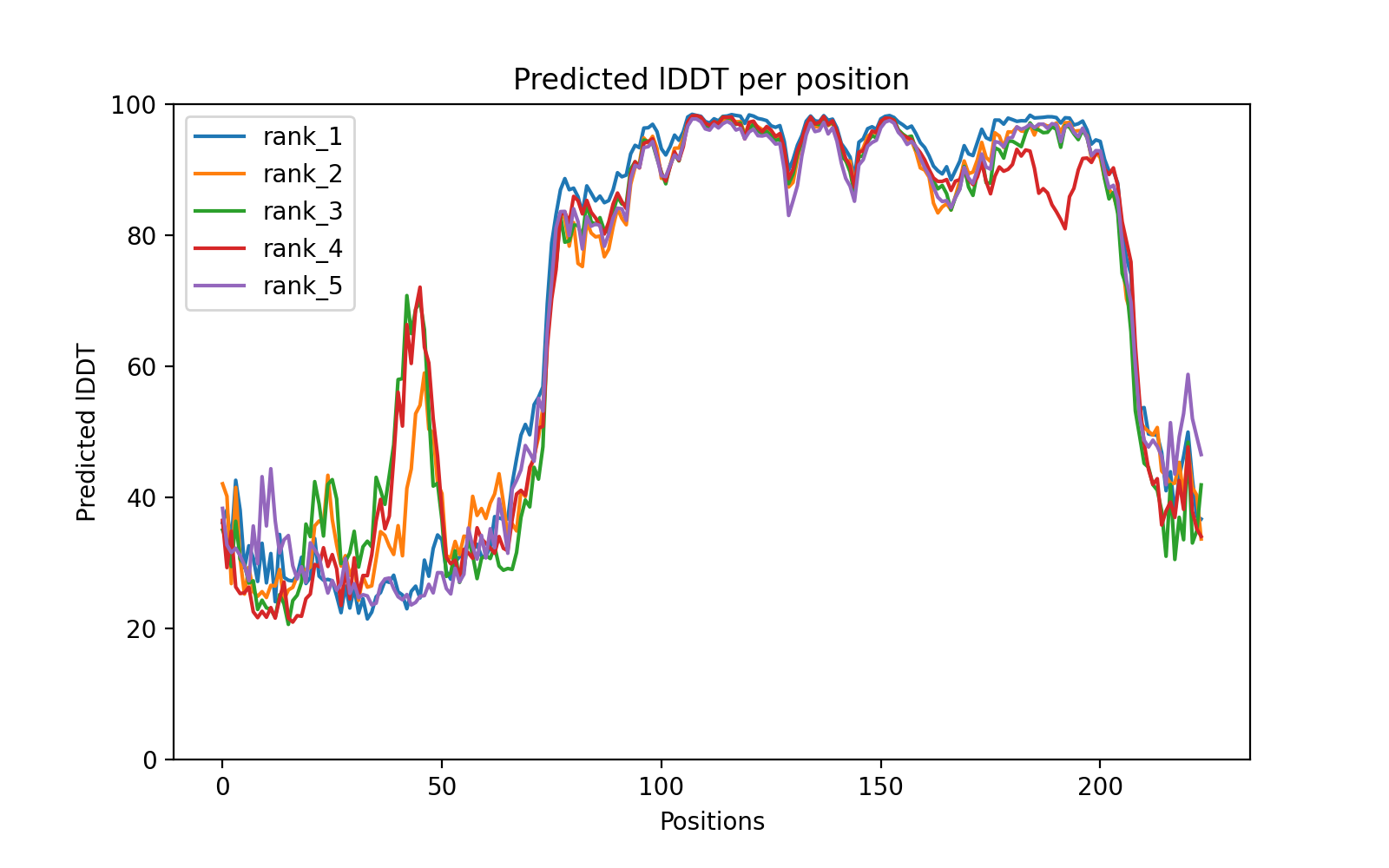

Supplement: Supplementary file 8 — Supplementary Data 5 [file 42003_2023_5501_MOESM8_ESM.zip › Supplementary Data 5/PCGF_putative_checks/PCGF_alphafold_remainedputative/PYGBIF005603_PCGF_458fe.result/PYGBIF005603_PCGF_458fe_plddt.png]

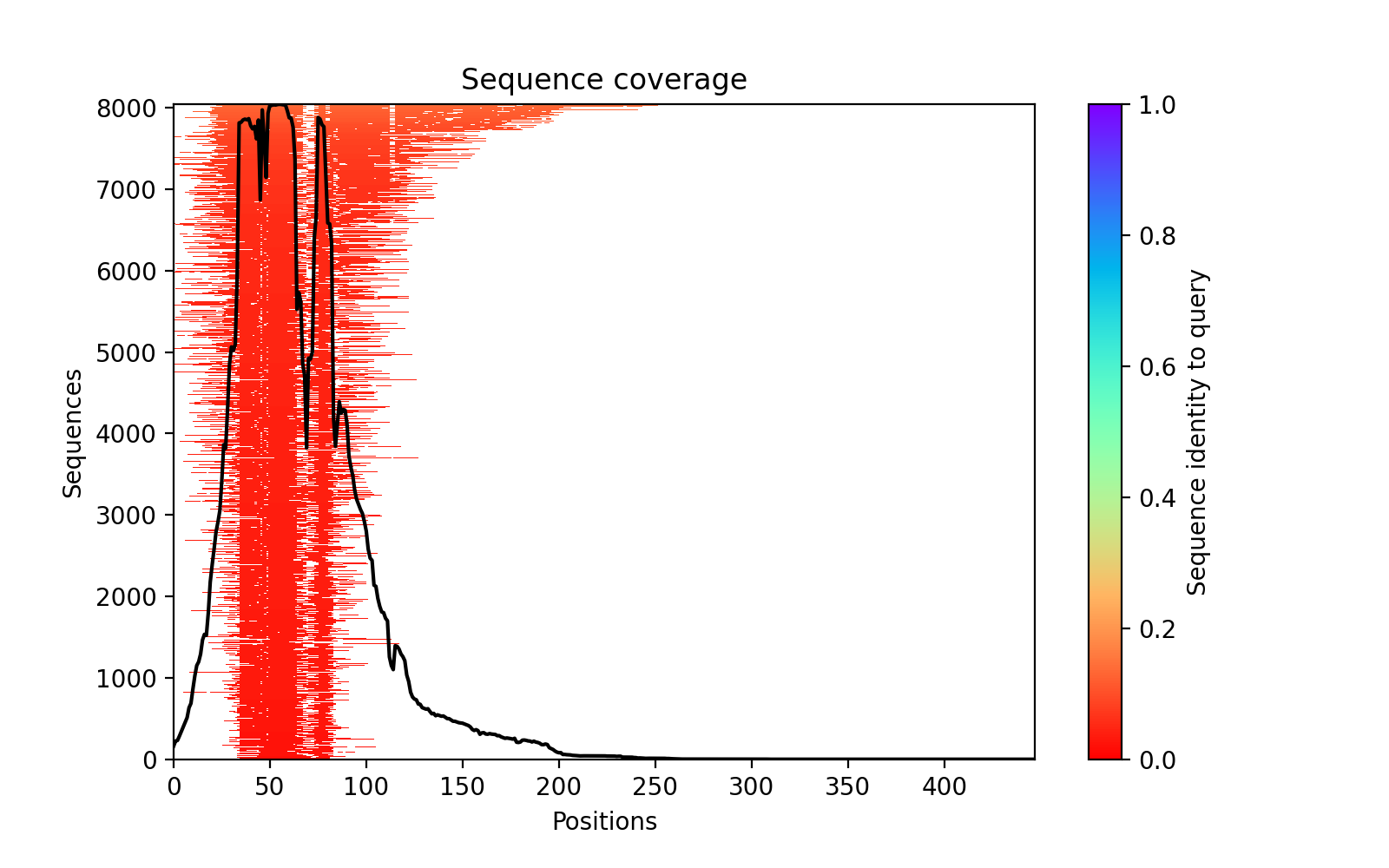

Supplement: Supplementary file 8 — Supplementary Data 5 [file 42003_2023_5501_MOESM8_ESM.zip › Supplementary Data 5/PCGF_putative_checks/PCGF_alphafold_remainedputative/TETTHE021998_PCGF_e59f4.result/TETTHE021998_PCGF_e59f4_coverage.png]

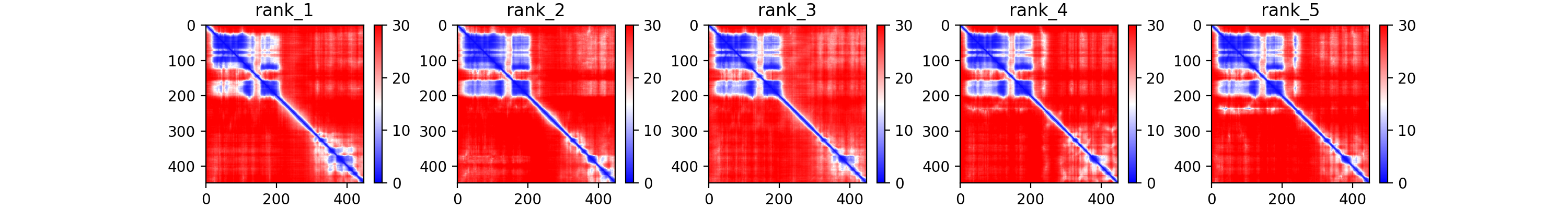

Supplement: Supplementary file 8 — Supplementary Data 5 [file 42003_2023_5501_MOESM8_ESM.zip › Supplementary Data 5/PCGF_putative_checks/PCGF_alphafold_remainedputative/TETTHE021998_PCGF_e59f4.result/TETTHE021998_PCGF_e59f4_PAE.png]

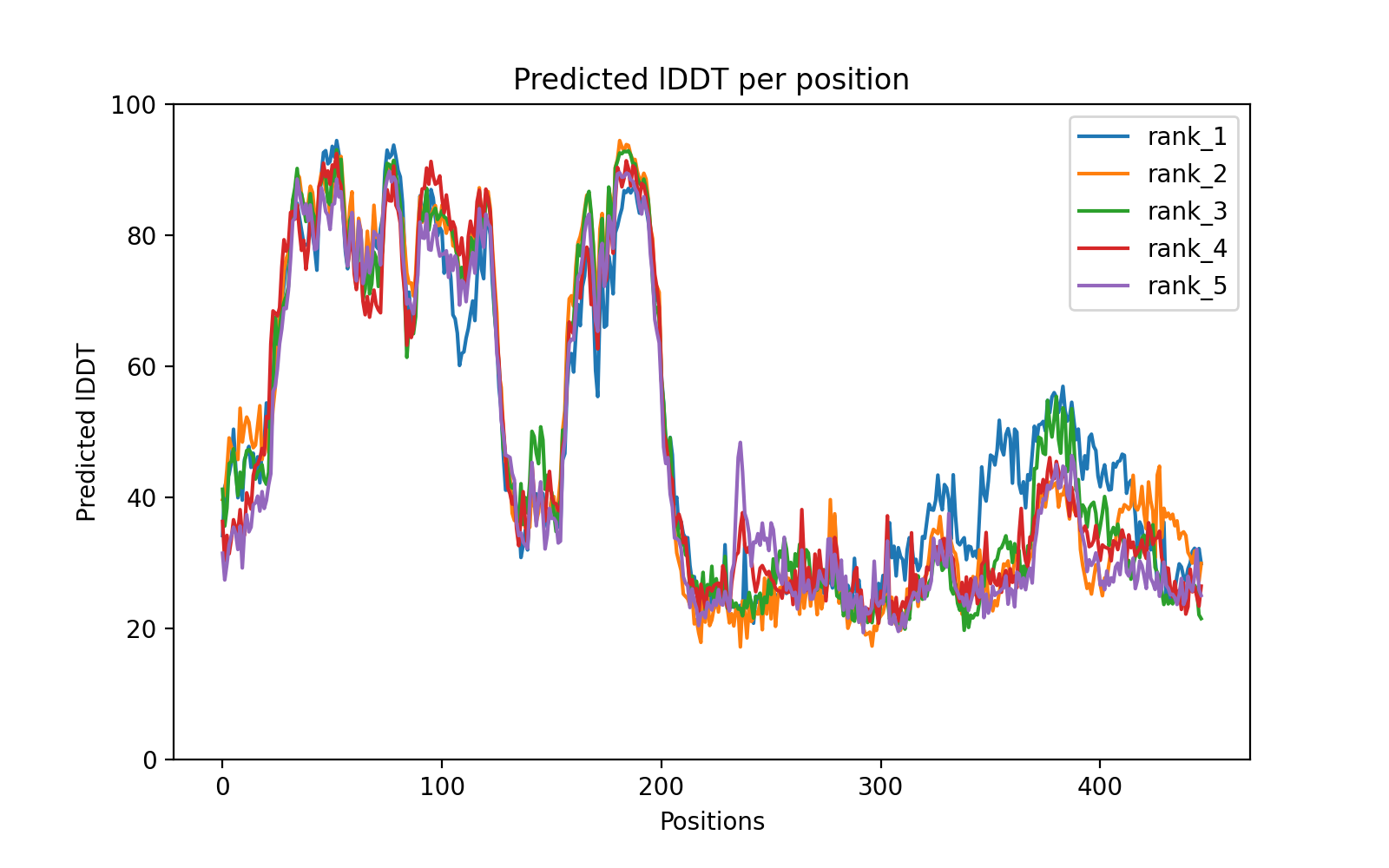

Supplement: Supplementary file 8 — Supplementary Data 5 [file 42003_2023_5501_MOESM8_ESM.zip › Supplementary Data 5/PCGF_putative_checks/PCGF_alphafold_remainedputative/TETTHE021998_PCGF_e59f4.result/TETTHE021998_PCGF_e59f4_plddt.png]

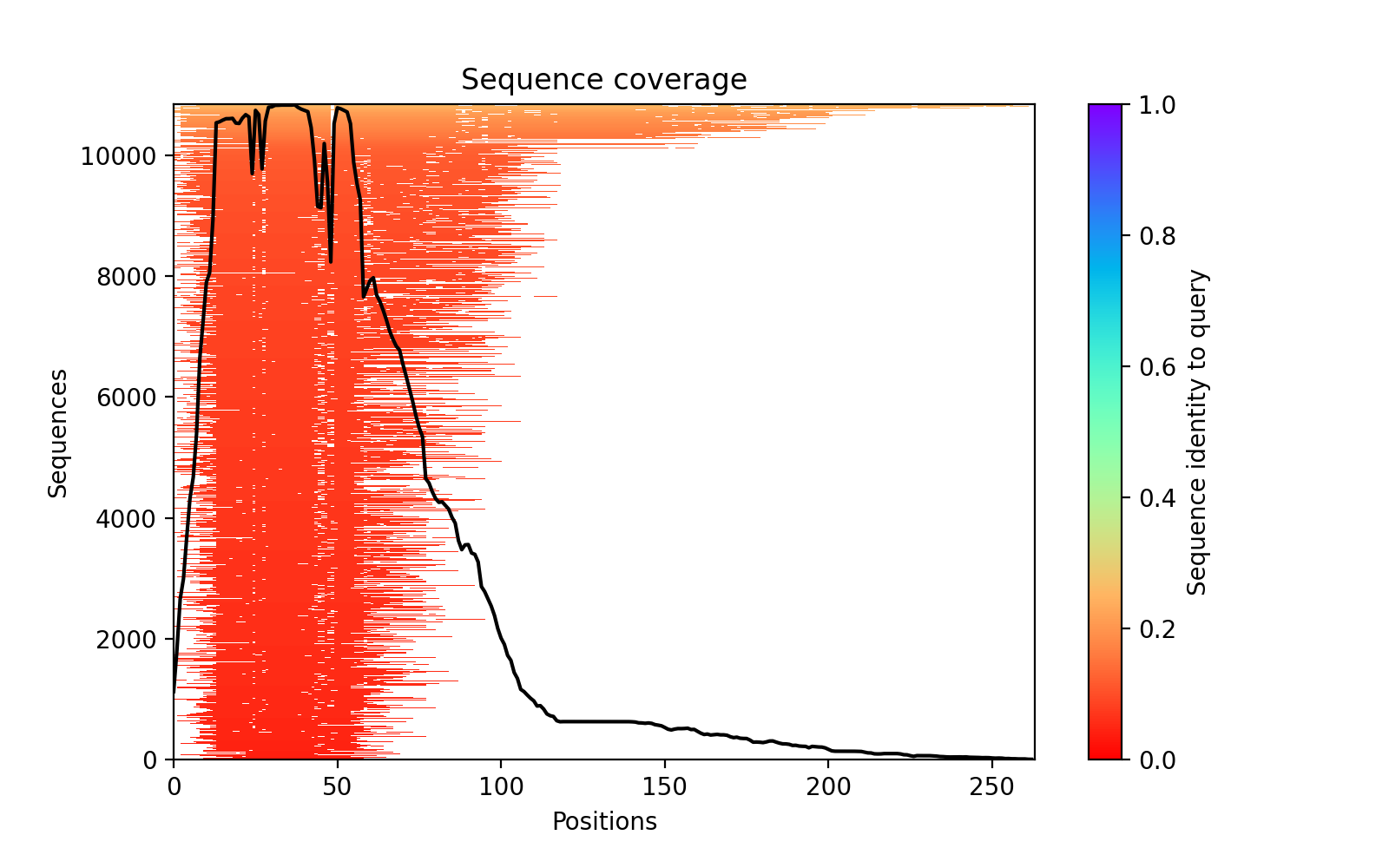

Supplement: Supplementary file 8 — Supplementary Data 5 [file 42003_2023_5501_MOESM8_ESM.zip › Supplementary Data 5/RING_putative_checks/RING_alphafold_remainedputative/COCSUB001834_RING_33e53.result/COCSUB001834_RING_33e53_coverage.png]

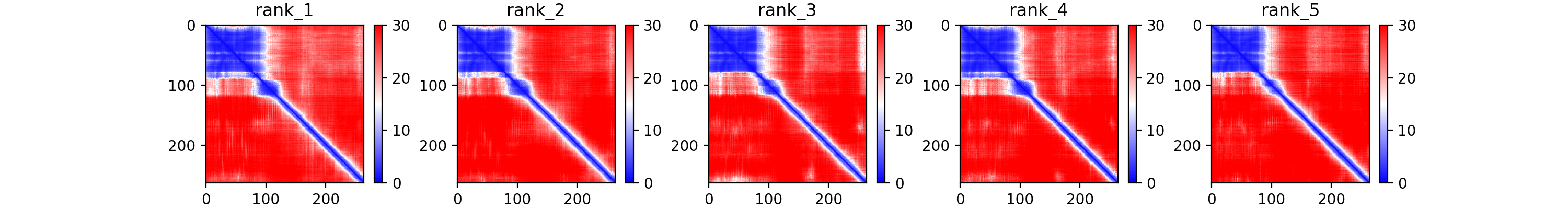

Supplement: Supplementary file 8 — Supplementary Data 5 [file 42003_2023_5501_MOESM8_ESM.zip › Supplementary Data 5/RING_putative_checks/RING_alphafold_remainedputative/COCSUB001834_RING_33e53.result/COCSUB001834_RING_33e53_PAE.png]

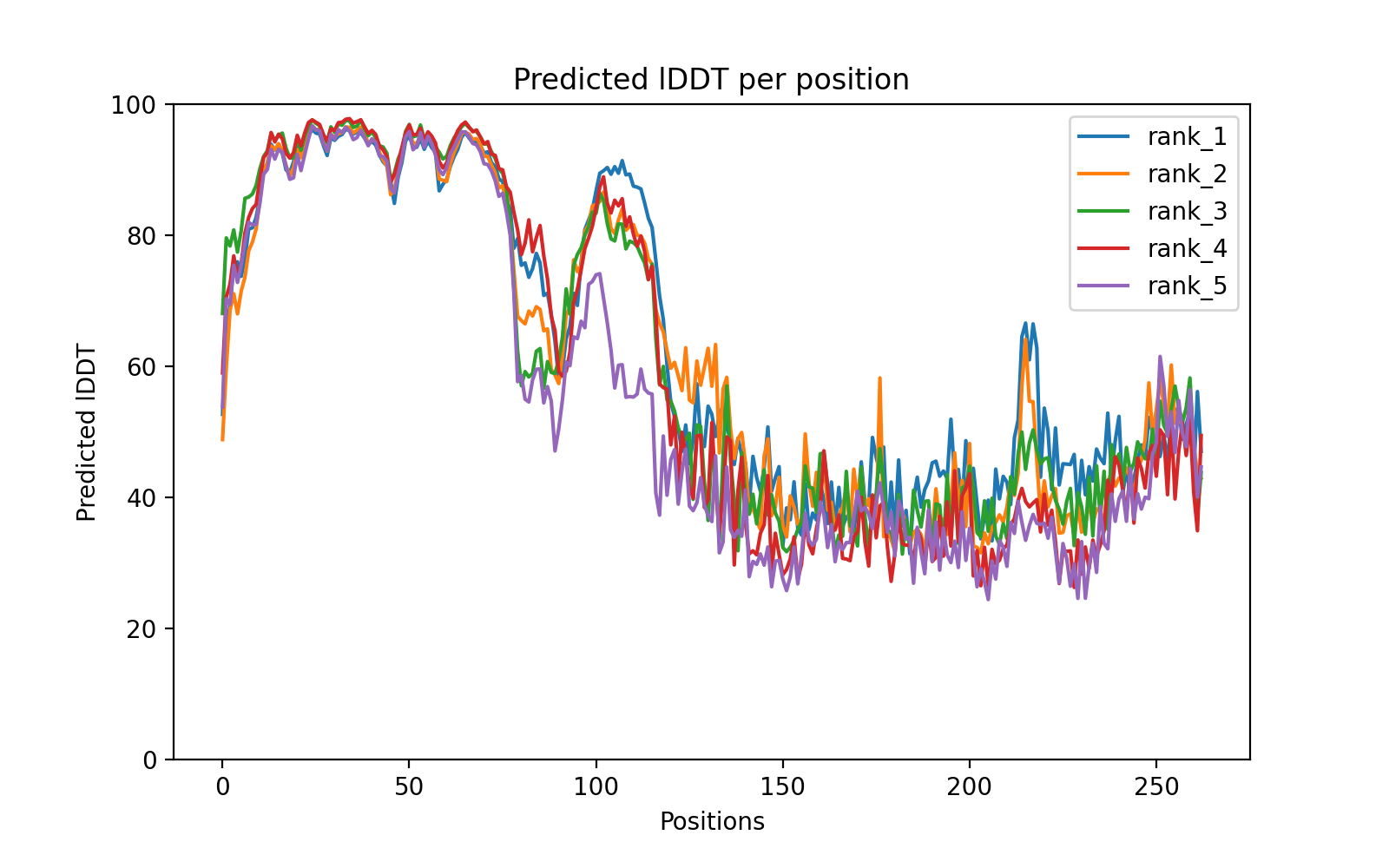

Supplement: Supplementary file 8 — Supplementary Data 5 [file 42003_2023_5501_MOESM8_ESM.zip › Supplementary Data 5/RING_putative_checks/RING_alphafold_remainedputative/COCSUB001834_RING_33e53.result/COCSUB001834_RING_33e53_plddt.png]
